# Altered Hepa1-6 cells by dimethyl sulfoxide (DMSO)-treatment induce anti-tumor immunity *in vivo*

**DOI:** 10.18632/oncotarget.7009

**Published:** 2016-01-25

**Authors:** Zhengyu Jiang, Hongxia Zhang, Ye Wang, Bin Yu, Chen Wang, Changcheng Liu, Juan Lu, Fei Chen, Minjun Wang, Xinlu Yu, Jiahao Lin, Xinghua Pan, Pin Wang, Haiying Zhu

**Affiliations:** ^1^ Department of Cell Biology, Second Military Medical University, Shanghai, P.R. China; ^2^ Center for Stem Cell and Medicine, The Graduate School, Second Military Medical University, Shanghai, P.R. China; ^3^ Department of Anesthesiology, Second Military Medical University, Shanghai, P.R. China; ^4^ Training Department, Second Military Medical University, Shanghai, P.R. China; ^5^ School of Clinic Medicine, Second Military Medical University, Shanghai, P.R. China; ^6^ Department of Genetics, Yale University School of Medicine, New Haven, CT, USA; ^7^ National Key Laboratory of Medical Immunology and Institute of Immunology, Second Military Medical University, Shanghai, P.R. China

**Keywords:** cancer immunotherapy, DMSO, anti-tumor immunity

## Abstract

Cancer immunotherapy is the use of the immune system to treat cancer. Our current research proposed an optional strategy of activating immune system involving in cancer immunotherapy. When being treated with 2% DMSO in culture medium, Hepa1-6 cells showed depressed proliferation with no significant apoptosis or decreased viability. D-hep cells, Hepa1-6 cells treated with DMSO for 7 days, could restore to the higher proliferation rate in DMSO-free medium, but alteration of gene expression profile was irreversible. Interestingly, tumors from D-hep cells, not Hepa1-6 cells, regressed in wild-type C57BL/6 mice whereas D-hep cells exhibited similar tumorigenesis as Hep1–6 cells in immunodeficient mice. As expected, additional Hepa1-6 cells failed to form tumors in the D-hep-C57 mice in which D-hep cells were eliminated. Further research confirmed that D-hep-C57 mice established anti-tumor immunity against Hepa1-6 cells. Our research proposed viable tumor cells with altered biological features by DMSO-treatment could induce anti-tumor immunity *in vivo*.

## INTRODUCTION

Cancer has become the leading cause of death with increasing cases worldwide [1]. As limited efficacy and considerable side effects burdening the application of traditional treatments, in recent years, immunotherapy has emerged as a promising approach for cancer treatment [2–6]. Activating immunization, as one of effective strategies has been developed for many years. Previous researches have been trying to target dendritic cells (DCs) [7–9], or taking the advantages of adjuvant [7, 10–11], virus [12–13] or specific peptide [14–16] to induce protective anti-tumor immunity. Especially, recent researches in altering the tumor microenvironment and depressing the immune-dampening effects, with multiple mechanism for establishing the immunity, showed affirmative outcomes [17–21]. Therefore, developing more approaches to induce protective anti-tumor immunity would extend the application of immunotherapy in cancer treatment.

As we know, DMSO (dimethyl sulfoxide) is an important amphipathic molecule that is widely used not only as a solvent for water-insoluble substances but also as a cryopreservant for cells. In addition, it is elucidated that DMSO can induce the differentiation of leukemia cells [22], activate tumor suppressor genes, induce apoptosis and inhibit the proliferation of various tumors [22–24]. Moreover, low concentrations of DMSO could lead to the change of genome-wide DNA methylation and hydroxymethylation profile as reported recently [25, 26]. However, no report was found on the biological feature *in vivo* or *ex vivo* of the DMSO-treated cells, from which the DMSO was removed after the treatment. In current research, we demonstrated that mouse hepatocellular carcinoma cell line, Hepa1-6 cells (Hep cells), when being treated with 2% vol DMSO, showed depressed proliferation and cell cycle arrest with no significant apoptosis or decreased viability. After DMSO was removed from medium, the proliferation of DMSO-treated cells was partially recovered and G_0_/G_1_ arrest was released. However, the alteration of gene expression profile has presented to be irreversible. The more interesting was that the altered cells, D-hep cells, Hep cells treated with DMSO for 7 days, could induce mice to establish anti-tumor immunity against Hep cells after being injected into wild type C57BL/6 mice. Thus, our research proposed the biological feature of tumor cells treated with DMSO and confirmed the establishment of anti-tumor immunity *in vivo* induced by D-hep cells. This may extend the potential applications of DMSO-treatment in cancer immunotherapy as an option to activate immune system against tumor cells.

## RESULTS

### DMSO inhibited the proliferation of Hepa1-6 but did not decrease the cell viability or induce apoptosis

**Figure 1:**
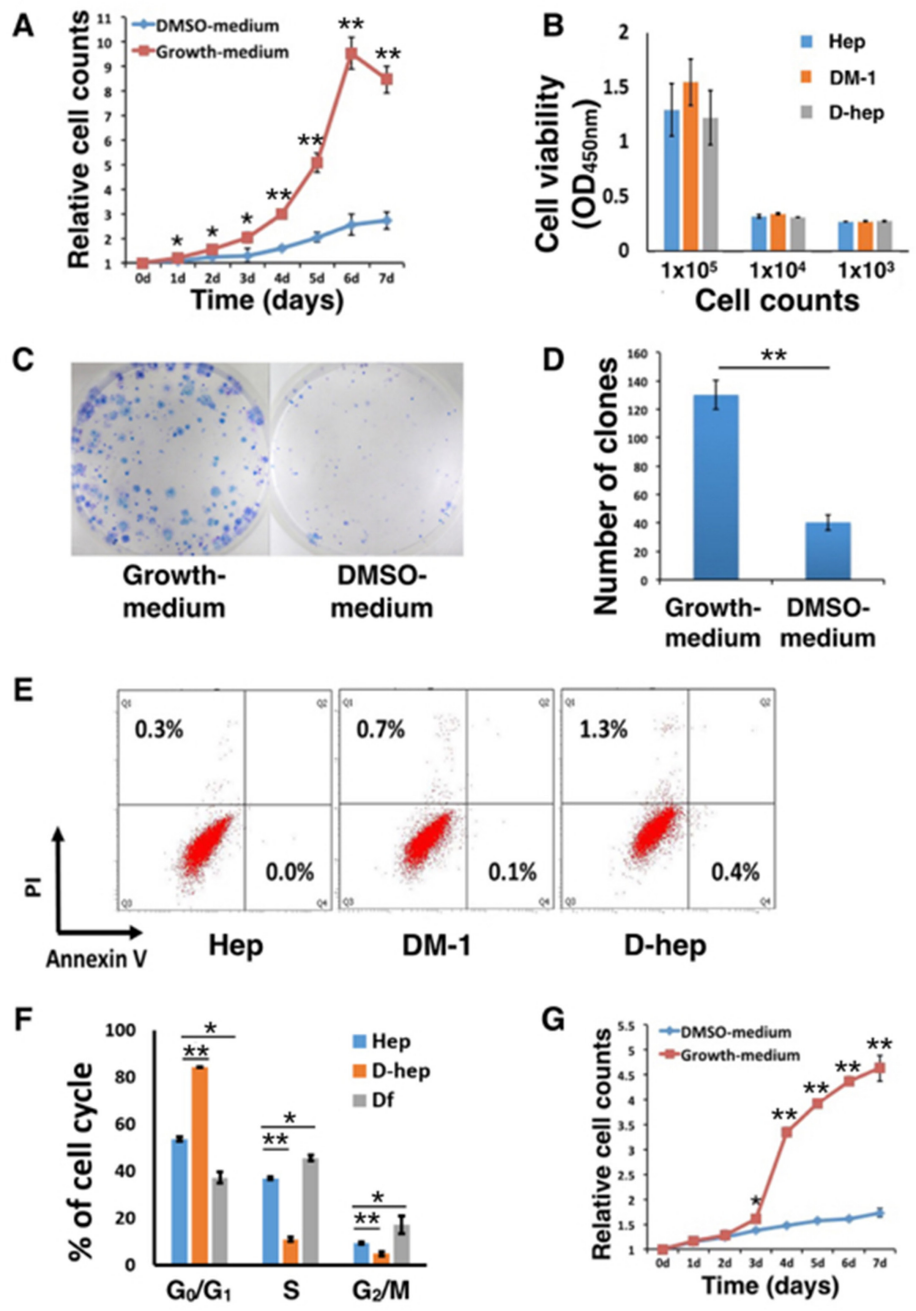
DMSO altered the *in vitro* proliferation ability and *in vivo* tumorigenicity of Hep cells (**A**) The proliferation rate, as analyzed by CCK-8 assays, of Hep cells was decreased in DMSO-medium (*n* = 10). (**B**) Cell viability of D-hep cells showed no differences with Hep cells (*n* = 6); (**C, D**) The colony-forming efficiency (CFE) of Hep cells was decreased in DMSO-medium compared with that in growth medium without DMSO (*n* = 6). (**E**) Apoptosis and necrosis of D-hep cells and Hep cells cultured in DMSO-medium for 1 day (DM-1) and 7 days (D-hep) showed no differences with Hep cells (*n* = 3). (**F**) Cell cycle analysis of Hep, D-hep and Df cells showed G_0_/G_1_ arrest when the cells were cultured in DMSO-medium and G_0_/G_1_ arrest released when cells were incubated in growth-medium (*n* = 3). (**G**) The proliferation rate of D-hep cells reached a higher level after the cells were cultured in growth medium than in DMSO medium (*n* = 10). The error bars represent ± S.D. (*P < 0.05, **P < 0.01); *n* = biological replicates.

The results from the CCK-8 assays showed that comparing with those cultured in growth medium, Hepa1-6 cells in DMSO-medium exhibited a decreased proliferation rate (Figure 1A), lower CFE (Figure 1C, 1D) and arrested cell cycle (Figure 1F) during 7-day incubation, but not decreased cell viability (Figure 1B) and increased apoptosis or necrosis (Figure 1E). After removing DMSO from medium in the following 7 days, D-hep cells could restore to higher proliferation rate (Figure 1G) than D-hep cells in DMSO-medium with the releasing of G_0_/G_1_ arrest (Figure 1F).

### Tumors derived from D-hep cells regressed in C57BL/6 mice but not in NOD/SCID or nude mice

**Figure 2:**
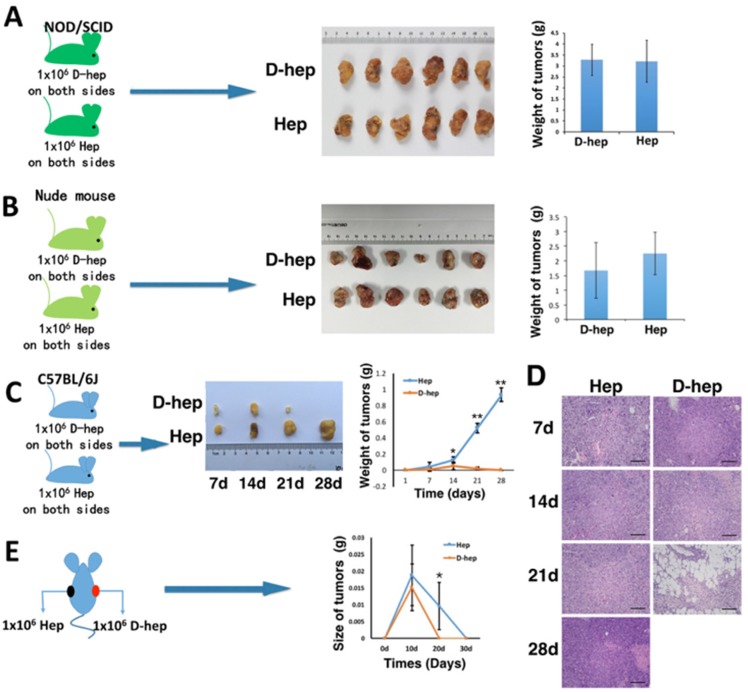
Tumorigenicity of Hep or D-hep cells in NOD/SCID mice, nude mice and C57BL/6 mice (**A**) No difference in the tumor mass between Hep tumors and D-hep tumors was observed after 4 weeks from injection in NOD/SCID mice (*n* = 6). (**B**) Both D-hep and Hep tumors could keep growing in nude mice 30 days from injection (*n* = 3). (**C**) The growth rate of D-hep tumors was lower than that of Hep tumors in WT-C57 mice, and the D-Hep tumors gradually regressed within 21 days (*n* = 3). Representative tumor tissues (C) and HE staining of Hep tumors and D-hep tumors (**D**) (Bar = 100 μm). (**E**) D-hep and Hep cells injection in each side of one mouse showed tumor regression represented by tumor size after 30-days from injection (*n* = 3). The error bars represent ± S.D. (*P < 0.05, **P < 0.01); *n* = biological replicates.

To investigate the tumorigenicity of D-hep cells, 1 × 10^6^ D-hep or Hep cells were suspended in 0.2 ml of PBS and subcutaneously injected into each side of inguen of NOD/SCID mice or nude mice. We observed that in NOD/SCID mice, both D-hep cell- and Hep cell-derived tumors, termed as D-hep tumor and Hep tumor respectively, kept growing during the four-week period and the final tumor masses were not significantly different (Figure 2A). In the same way, both D-hep tumors and Hep tumors could form and grow successfully in nude mice in 30-day (Figure 2B, Supplementary Figure 1).

However, the tumorigenicity of D-hep or Hep cells were much more different from each other in wild-type C57BL/6 mice (WT-C57). After the same amount of D-hep cells and Hep cells were injected into C57BL/6 mice, during the first two weeks after injection, tumor formation and growth were observed, though D-hep tumors were smaller than Hep tumors. After that, at the third week after injection, the D-hep tumors have been soft and smaller gradually whereas the Hep tumors kept growing. And in the forth week, D-hep tumors almost regressed and eliminated while Hep tumors grew much bigger (Figure 2C). The mice undergoing growth and regression of D-hep tumors were termed as D-hep-C57 mice. We harvested the tumors tissues on day 7, 14, 21 and 28 after injection and confirmed, by haematoxylin and eosin (HE) staining, that true tumor tissues, not inflammatory pseudotumors or focal fibroses, had formed or regressed during the four-week period (Figure 2D). Moreover, we injected 1 × 10^7^ D-hep cells into WT-C57 mice to further observe tumorigenesis of D-hep. As expected, even though the cell number was increased by 10 times, the D-hep tumors still underwent formation firstly, regression gradually and elimination finally (Supplementary Figure 2) during four weeks, which suggested D-hep cells had no risk of tumorigenesis *in vivo*. Besides, we also observed there was no tumor formation when additional 1 × 10^7^ Hep cells were injected into D-hep-C57 mice whereas Hep-tumors continued growing in WT-C57 mice in four weeks (Supplementary Figure 3).

Interestingly, when D-hep cells were injected on one side of inguen and Hep cells were injected on the other side in the same mouse, not only D-hep tumors but also Hep tumors formed in 10 days and regressed gradually after that. Both kinds of tumor were eliminated 30 days after the injection. The difference in tumorigenesis between them was Hep cells could form bigger tumors than D-hep cells during 10 days from injection and regressed later and slower than D-tumors, which were shown as the tumor size on day 10, 20 and 30 (Figure 2E).

### Effector T cells and NKT cells were activated in C57BL/6 mice by D-hep cells

**Figure 3:**
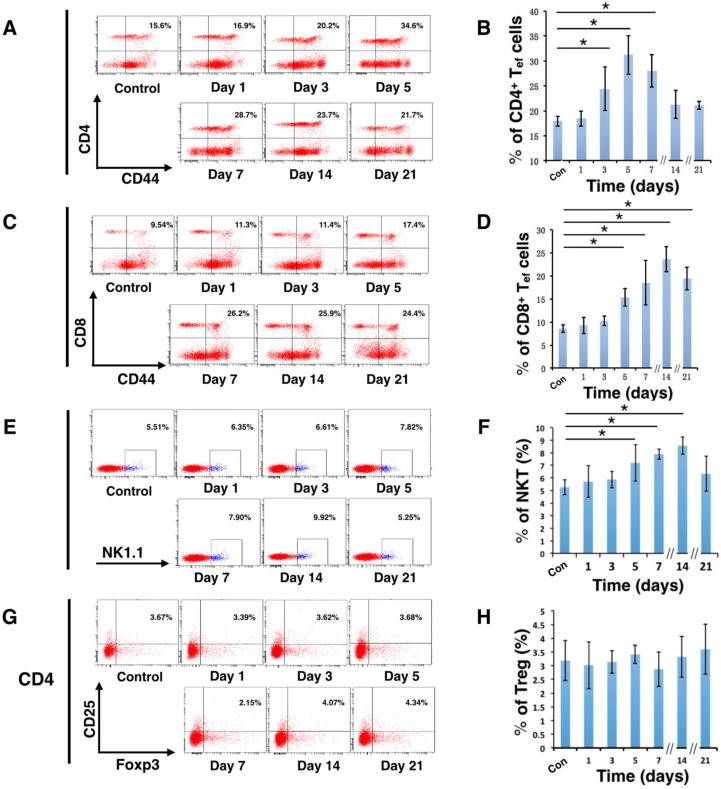
D-hep cells activated effector T cells and NKT cells in C57BL/6 mice Representative flow cytometry dot plots and graphs illustrated the percentage change of T cells before (Con) and after D-hep cell challenge (days 1, 3, 5, 7, 14, 21). (**A–D**) Representative flow cytometry dot plots and graphs illustrated that CD4^+^ effector T cells (CD4^+^CD44^+^) were activated on day 3 and that CD8^+^ effector T cells (CD8^+^CD44^+^) were activated on day 5, with continuous increases until day 14 (*n* = 3). (**E–F**) Representative flow cytometry dot plots and graphs of NKT cells (NK1.1^+^) showed that the NKT cells continued increasing from day 5 until day 14 (*n* = 3). (**G–H**) Representative flow cytometry dot plots and graphs of T_reg_ cells (CD4^+^CD25^+^Foxp3^+^) showed no significant changes before or after D-hep cell injection (*n* = 3). The error bars represent ± S.D. (*P < 0.05); *n* = biological replicates.

**Figure 4:**
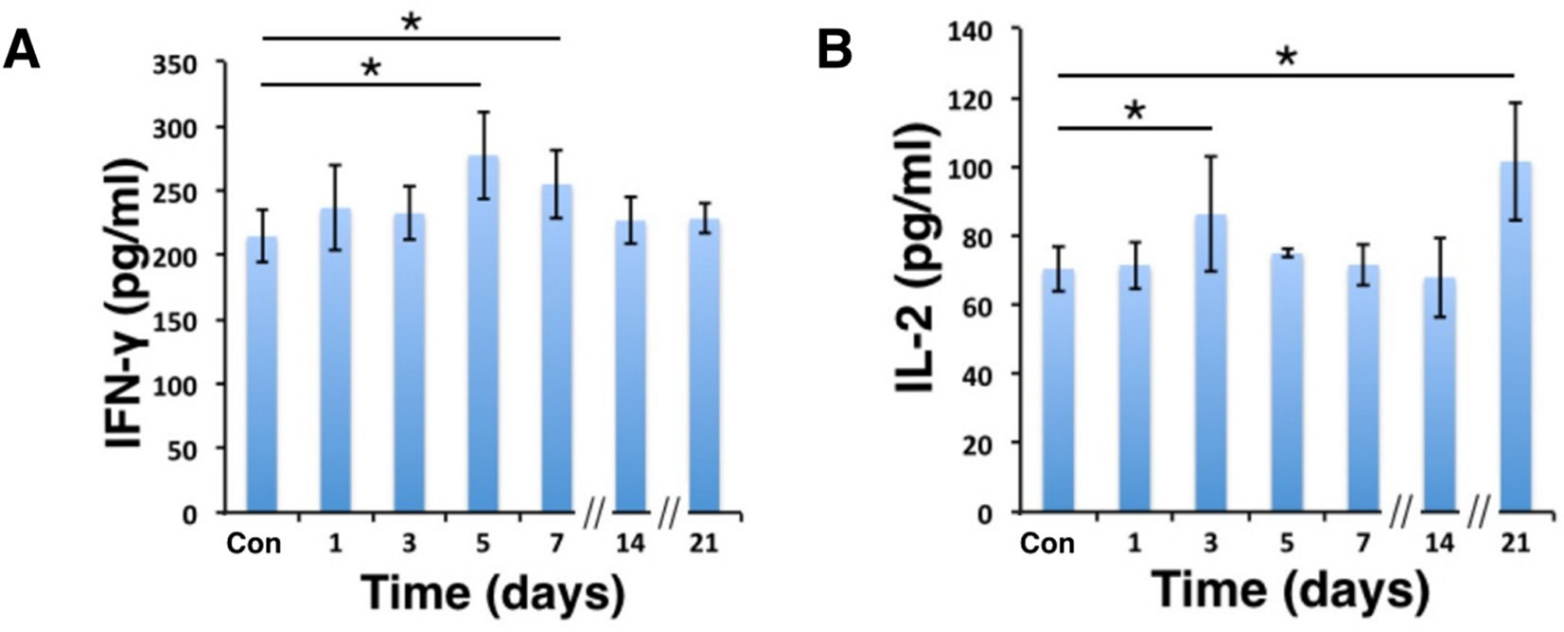
IFN-γ and IL-2 levels were elevated in serum after D-hep cell challenge The concentrations of IFN-γ (**A**) and IL-2 (**B**) in serum were analyzed using ELISA assay was shown before (Con) and after D-hep cell injection (days 1, 3, 5, 7, 14, 21). IFN-γ reached its highest level on day 5 whereas IL-2 did so on day 21 (*n* = 3). The error bars represent ± S.D. (*P < 0.05); *n* = biological replicates.

**Figure 5:**
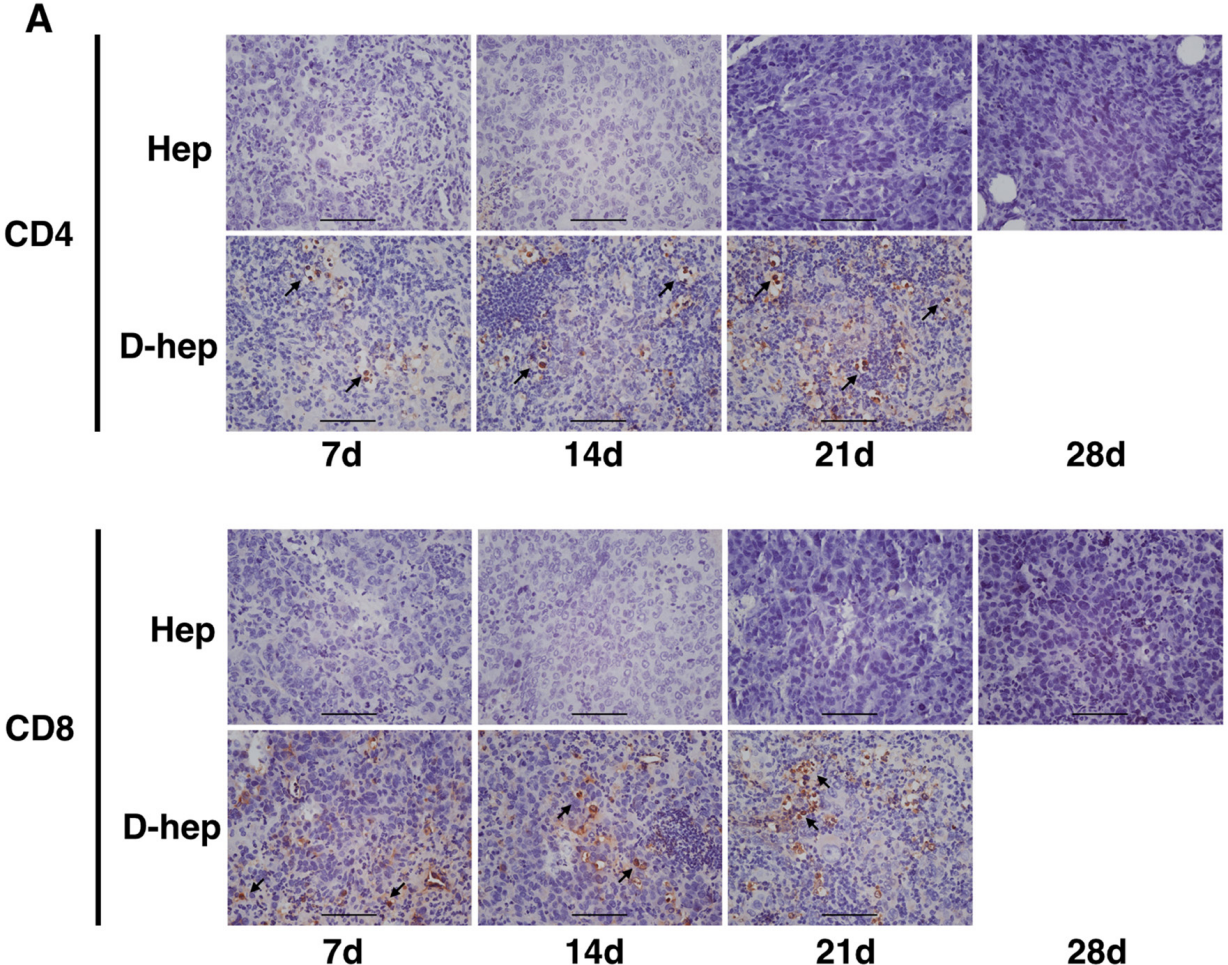
An increasing number of tumor infiltrating lymphocytes (TILs) in CD4^+^ and CD8^+^ subsets were found in D-hep tumors while few TILs were found in Hep tumors (Bar = 100 μm) Arrow showed TILs in each subsets

To further clarify whether the immune system participated in the tumor regression of D-hep cells, we next analyzed several subsets of lymphocytes and cytokines that could contribute to tumor regression. As shown in Figure 2A–2D, compared with mice injected with PBS, the percentage of CD4^+^ effector T cells (CD4^+^CD44^+^) increased on day 3 (Figure 3A, 3E) after injection. Following that, CD8^+^ effector T cells (CD8^+^CD44^+^) and NKT cells (NK1.1^+^) (Figure 3C, 3D) began expanding on day 5 and reached their highest level on day 14 (Figure 3B, 3E) at which D-hep-tumors began to regress. However, the number of T_reg_ cells (CD4^+^CD25^+^Foxp3^+^) was not significantly altered after D-hep cells were injected into WT-C57 mice (Figure 3F, 3H) compared with the control group. Moreover, because the activation of lymphocytes is usually accompanied by rising level of IFN-γ and IL-2 in serum [28], which triggers a positive feedback loop for NKT cell expansion and the cytotoxic activity of CD8^+^ effector T cells [28, 29], we also assessed the concentrations of IFN-γ and IL-2 in serum. As expected, the amount of IFN-γ increased by about 30% by day 5, and the amount of IL-2 increased by about 40% by day 21 (Figure 4A, 4B) in D-hep-C57 mouse serum, which was in accordance with the expansion of NKT cells and CD8^+^ effector T cells. In addition, in order to prove the effector T cells could be recruited into tumor site and execute anti-tumor immunity, immunohistochemistry was performed to analyze tumor infiltrating lymphocytes (TILs). As the results showed (Figure 5A), an increasing number of both CD4^+^ and CD8^+^ lymphocytes subsets were found on day 7, day 14 and day 21 in D-hep-tumors while few lymphocytes were found in Hep-tumors. Collectively, these results elucidated that D-Hep cells activated the immune system involving in the regression of D-hep tumors

### Memory and effector T cells were activated in D-hep-C57 mouse by Hepa1-6 cells

We sought to further verify whether D-hep-C57 mice had indeed acquired tumor-specific immunity and long-term memory against Hep cells. Thus, we subcutaneously injected 1 × 10^7^ Hepa1-6 cells on each side of the inguen of D-hep-C57 mice to observe whether the Hep cells could form tumors. In order to confirm the establishment of anti-tumor immunity, we detected the changes in the proportion of the T cell subset in spleen by flow cytometry and the concentration of IFN-γ and IL-2 in serum by ELISA after Hep challenge. WT-C57 mice injected with 1 × 10^7^ Hep cells were used as control group.

**Figure 6:**
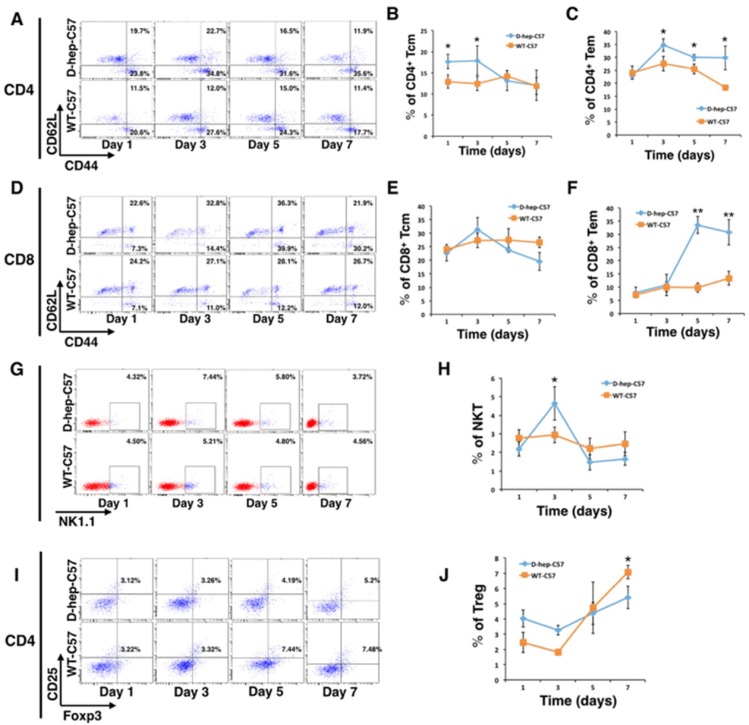
Memory and effector T cells were activated after Hep cells were injected into D-hep-C57 mice Flow cytometry analysis of CD4^+^ central memory T cells (CD4^+^CD44^high^CD62L^high^), CD4^+^ effector memory T cells (CD4^+^CD44^high^CD62L^low^), CD8^+^ central memory T cells (CD8^+^CD44^high^CD62L^high^), CD8^+^ effector memory T cells (CD8^+^CD44^high^CD62L^low^), NKT cells (NK1.1^+^) and regulatory T (T_reg_) cells (CD4^+^CD25^+^Foxp3^+^). Representative flow cytometry dot plots and graphs illustrated the percentage change of T cells on days 1, 3, 5 and 7 after injection (*n* = 3). (**A–C**) Representative flow cytometry dot plots (A) and graphs illustrated that CD4^+^ memory T cells were activated on day 1 after Hep cell injection and maintained this high level until day 3 (B). This activation was followed by CD4^+^ effector memory T cell activation from day 3 until day 7 (C). (D-F) Representative flow cytometry dot plots (**D**) and graphs illustrated that no significant changes in CD8^+^ memory T cells (**E**) occurred but that a distinct elevation in CD8^+^ effector memory T cells on day 5 was observed that increased up to 7 fold (**F**) after Hep cell injection. (**G–H**) Representative flow cytometry dot plots (**G**) and graphs illustrated that NKT cells were activated on day 3 after Hep cell injection (H). (**I–J**) Representative flow cytometry dot plots (I) and graphs illustrated that the number of T_reg_ cells continued increasing in WT-C57 mice but that no significant change was observed in D-hep-C57 mice after Hep cell injection (J). The error bars represent ± S.D. (*P < 0.05, **P < 0.01); *n* = biological replicates.

**Figure 7:**
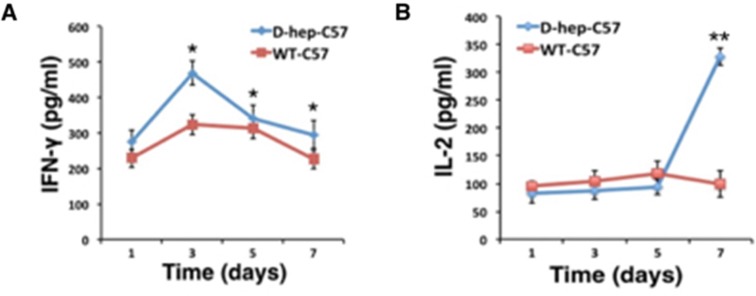
IFN-γ and IL-2 levels were increased in D-hep-C57 mice after Hep cell challenge The concentration of IFN-γ (**A**) and IL-2 (**B**) in serum on days 1, 3, 5 and 7 in WT-C57 or D-hep-C57 mice was shown after Hep cell challenge. IFN-γ reached its highest level on day 3, whereas IL-2 did so on day 7 (*n* = 3). The error bars represent ± S.D. (*P < 0.05, **P < 0.01) *n* = biological replicates.

Results showed that the percentage of CD4^+^ central memory T cells (CD4^+^CD44^high^CD62L^high^) increased on day 1 and was maintained at a high level until day 3 after injection. Subsequently, the CD4^+^ effector memory T cells (CD4^+^CD44^high^CD62L^low^) reached their highest level and were maintained at a higher percentage than the WT-C57 group at the day 3, 5 and 7 (Figure 6A–6C). Although there was no significant change in the number of CD8^+^ central memory T cells (CD8^+^ CD44^high^CD62L^high^) (Figure 6D, 6E), a distinct activation of CD8^+^ effector memory T cells (CD8^+^CD44^high^ CD62L^low^) appeared on the day 5 after injection (Figure 6D, 6F) that reached a level that was 3-fold higher than that of WT-C57 mice on the same day and was maintained till day 7. Meanwhile, the number of NKT cells was increased 2.5-fold compared with the WT-C57 group on day 3, but decreased to a normal level by day 5 (Figure 6G, 6H). By contrast, there was no statistics difference in percentage of T_reg_ cells between two groups (Figure 7I, 7J). Moreover, an increased amount of IFN-γ and IL-2 in the serum could be detected on days 3 and day 7 (Figure 7A, 7B), and cytokines reached their highest levels in serum earlier, with higher concentration, than that in the first injection of D-hep cells. Collectively, these results indicated that the D-hep-C57 mice established anti-tumor immunity and long-term memory T cells against Hepa1-6 cells.

### Splenic T cells can be activated and induce cytotoxicity when co-cultured with Hepa1-6 cells *in vitro*

**Figure 8:**
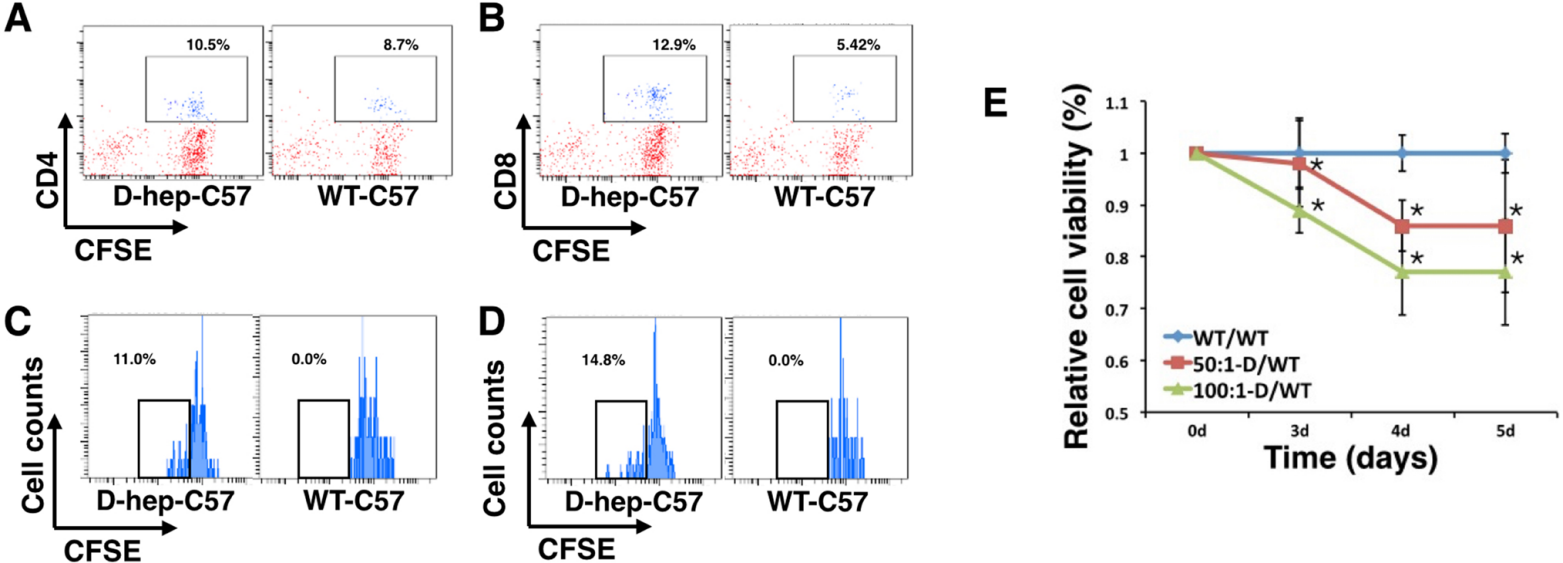
Lymphocytes from D-hep-C57 mice were activated by Hep cells and induced cytotoxicity *in vitro* (**A–B**) Flow cytometry analysis of CD4^+^ and CD8^+^ T cells in co-culture systems of Hep cells and splenocytes from WT-C57 or D-hep-C57 mice after 3 days of culturing illustrated that lymphocytes from D-hep-C57 mice showed a higher percentage of CD4^+^ and CD8^+^ T cells compared with those from WT-C57 mice. (**C–D**) Flow cytometry analysis of dividing CD4^+^ and CD8^+^ T cells, as shown by CFSE detection in co-culture systems of Hep cells and splenocytes from WT-C57 or D-hep-C57 mice after 3 days of culturing, illustrated that only lymphocytes from D-hep-C57 mice showed reduced fluorescence intensities of 11.0% and 14.8% for CD4^+^ (C) and CD8^+^ (D) T cells respectively. (**E**) The relative cell viability of Hep cells in the co-culture system was analyzed using CCK-8 assays. Splenocytes from D-hep-C57 (D-group) or WT-C57 (WT-group) mice were co-cultured with Hepa1-6 cells for 3, 4 or 5 days respectively at effector-target ratios of 50:1 or 100:1 (*n* = 6). The error bars represent ± S.D. (*P < 0.05); *n* = biological replicates.

Splenocytes from D-hep-C57 mice or WT-C57 mice were collected and co-cultured with Hep cells at a ratio of 50:1 or 100:1. The splenocytes were labled with CFSE previously. 3 days later, the splenocytes were stained with CD4^+^ and CD8^+^ antibodies for flow cytometry. The results showed that the expansion of T cells in D-hep-C57 mice was higher than that in WT-C57 mice for both the CD4^+^ and CD8^+^ T cell subsets (Figure 8A, 8B). In addition, splenocytes from D-hep-C57 mice presented high frequencies of dividing CD4^+^ and CD8^+^ T cell subsets, which were increased by 11% in CD4^+^ T cells and 14.8% in CD8^+^ T cells, whereas no reduced fluorescence intensity was observed in splenocytes from WT-C57 mice (Figure 8C, 8D). In the meantime, cytotoxicity effects of activated lymphocytes to Hep cells were assessed through cell viability. CCK-8 assay results exhibited that tumor cells co-cultured with splenocytes from D-hep-C57 mice showed decreased cell viability after 3-day-incubation in an effector-target ratio- or time-dependent manner (Figure 8E). Taken together, the results indicated that splenocytes from D-hep-C57 mice could be activated by Hep cells and presented cytotoxicity to tumor cells antigens *in vitro*, which suggests that the immunosurveillance and long-term memory against Hepa1-6 tumor antigens are well established in D-hep-C57 mice.

### D-hep mouse showed capacity to suppress tumorigenesis of mouse melanoma cell line B16-F10

**Figure 9:**
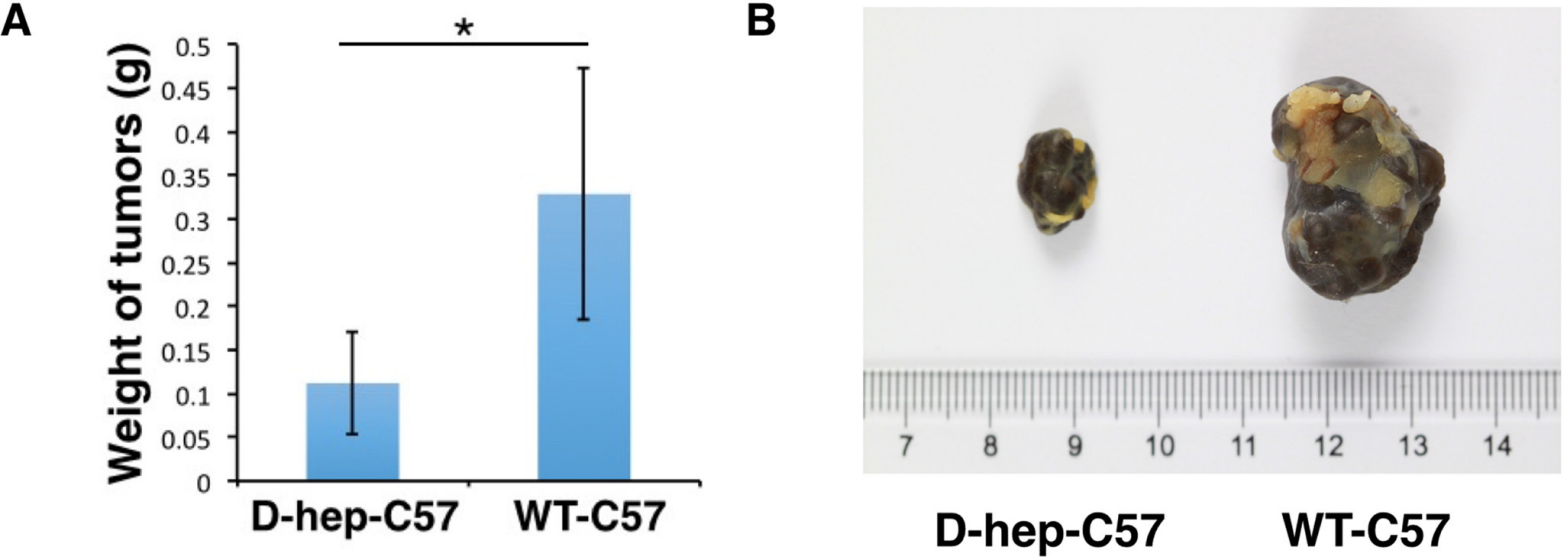
D-hep-C57 mice showed the ability to depress tumourigenicity of the mouse melanoma cell line B16-F10 cells (**A**) Weight graph and (**B**) representative tumors derived from 1 × 10^5^ B16-F10 cells injected into the subcutaneous inguen of D-hep-C57 and WT-C57 mice (***n =*** 6) was shown. The error bars represent ± S.D. (*P < 0.05); *n* = biological replicates.

To investigate whether D-hep mice can suppress other tumor growth, we furthered experiments by injecting other kinds of tumor cells including mouse prostate tumor cell line RM-1, mouse ascites hepatoma cell line H22 and mouse melanoma cell line B16-F10 which are all derived from C57BL/6 mouse. Though D-hep mice showed no capacity to suppress tumor growth of H22 and RM-1, B16-F10 tumors were highly depressed in D-hep mice (Figure 9A, 9B), indicating the anti-tumor immunity induced by D-hep cells could also suppress the growth of B16-F10-derived tumors.

## DISCUSSION

Existing researches have elucidated the function of DMSO in inducing cell differentiation [22], suppressing proliferation and inducing apoptosis [22–24]. Recent research also pointed out DMSO could change genome-wide DNA methylation and hydromethylation pattern by influencing the expression of DNA methyltransferases (Dnmt1, Dnmt3a and Dnmt3b) and TET (ten-eleven translocation proteins) family [25, 26]. However, researches mentioned above mainly focused on the status of cells incubated in DMSO-medium *in vitro.* Here, we reported the influence of DMSO on biological characteristics of Hepa1-6 cells *ex-vivo* and *in vivo* in comprehensive way. First of all, DMSO didn't decrease the viability or induce apoptosis of Hep cells. We speculate that this may result from the different concentration of DMSO used in experiments or the difference in tolerance or sensitivity towards DMSO among cell lines. Also, this result helps to eliminate the possibility that a large amount of dead or dying D-Hep cells due to growth-disadvantage lead to the lower proliferation *in vitro* and lower tumorigenicity in WT-C57 mice, or possibilities that antigens exposed in dead or dying cells are recognized by mouse immune system. Secondly, DMSO-treatment confers D-Hep cells different properties, which presented as a recovered higher level of proliferation in growth-medium and different gene expression profile. The initiative analysis by gene expression array showed that about more than 1000 genes were altered by DMSO-treatment (Supplementary Table 1). In further investigation, we selected a part of altered genes and detected their expression in Hep cells, D-hep cells and Df cells by real-time PCR. The results exhibited that the expression pattern of selected genes in D-hep cells and Df cells has been changed compared with Hep cells, meanwhile, to a great extent, the change of gene expression profile in Df cells kept pace with that in D-hep cells (Supplementary Figure 4). This suggests that DMSO-treatment brings an irreversible change to Hep cells. Though the specific mechanisms about the alteration still remain unclear, this permanent alteration might be important clue to explore how D-hep cells induce anti-tumor immunity *in vivo*.

The difference of tumorigenesis between D-hep and Hep cells *in vivo* is also an evidence on the alteration of biological features resulting from DMSO-treatment. The current data showed that D-Hep tumors underwent the tumor formation, regression and elimination in WT-C57 mice but formed tumors in the same way as Hep cells in immunodeficient mice. Considering either NOD/SCID mice or nude mice are deficient in T lymphocytes, we tend to believe that T lymphocytes, as major subset, mediated the anti-tumor immunity, which is also evidenced by the sequential expansion of T cell subsets, specific T cells proliferation and cytotoxicity assay.

To our knowledge, the effects of DMSO treatment on tumor cells *in vivo*, such as inducing tumor-specific anti-tumor immunity, have never been reported. In our study, we observed the sequential activation of CD4^+^ effector T cells, NKT cells and CD8^+^ effector T cells. It is well known that CD8^+^ effector T cells is a key subset in effective anti-tumor immunity [30]. And recent researches uncovered that, as an immunoregulatory population, NKT cells could participates in antigen recognition, induce the maturation of CD8^+^ effector T cells and DCs in immune responses and thus induce innate and adaptive anti-tumor immunity [28, 31, 32]. Besides, it has been reported that the activation and activity of NKT and CD8^+^ T cells are related to the increasing secretion of IFN-γ [28, 33–35], which has also been exhibited between NKT and IFN-γ in a positive correlation as Mattarrolo [28] reported. Therefore, the results above revealed lymphocyte activation and NKT cell responses may contribute to inducing anti-tumor immunity after D-Hep cells injection. Moreover, the activation of T_cm_ and T_em_ in secondary response that was induced by Hep cells also exhibited tumor-specific anti-tumor immunity has been well established in D-hep-C57 mice. T_cm,_ as a subset in both CD4^+^ and CD8^+^ T cells with capacity of self-renewal and differentiation, was thought to mediate recall responses and expand before T_em_, while T_em_ acquiring more proliferation potential could mediate protective immune responses by accessing the tissue sites [36–38], In our experiments, we observed a rapid expansion of CD4^+^ T_cm_ within one day after Hep cells injection, which was followed by a 3-fold increase in CD8^+^ T_em_ levels. And also we observed the infiltrating CD4^+^ and CD8^+^ in D-hep-tumors. Combing with the results that co-culturing splenocytes and tumor cells leading to activation of T cells and cytotoxicity *in vitro*, we believe the anti-tumor immunity was memorized, which could be activated and eliminated tumor cells when encountering the same tumor-associated antigens.

A more interesting phenomena is that when Hep cells and D-Hep cells were injected into the same mouse, either Hep-tumor or D-Hep tumor could form initially but eliminate in the end, especially Hep-tumors regressed following D-Hep-tumors. These results were in accordance with the results of lymphocytes activation induced by D-hep cells and suggested the further therapeutic use of DMSO-treated tumor cells to trigger anti-tumor immunity.

Notwithstanding the immunity against Hepa1-6 cells, the D-hep-C57 mice also showed a capacity to depress the growth of the other types of tumor. We observed that when the mouse melanoma cell line B16-F10 cells were injected into D-hep-C57 mice, the growth and final weight of tumors were significantly decreased compared with that in WT-C57 mice. These results provide indications for exploring the mechanism of anti-tumor immunity induction and expand the potential for DMSO treatment as an optional strategy for cancer immunotherapy.

In summary, our research proposes the biological feature of tumor cells treated with DMSO and confirmed the ability of D-hep cells to induce anti-tumor immunity *in vivo*, which suggests the possible applications of DMSO-treatment in tumor immunotherapy.

## MATERIALS AND METHODS

### Cell culture and mice

Mouse hepatocellular carcinoma cell line Hepa1-6, mouse melanoma cell line B16-F10, mouse prostate tumor cell line RM-1 and mouse ascites hepatoma cell line H22 were originally purchased from Cell Bank of the Type Culture Collection (Chinese Academy of Sciences, Shanghai, China) and were maintained in the Department of Cell Biology, SMMU. Cells were cultured in complete DMEM (Hyclone, USA) or RPMI 1640 (Hyclone, USA) supplemented with 10% fetal bovine serum (FBS, Gibco, USA) and 1% 100 U/ml penicillin-streptomycin (Hyclone, USA). D-hep cells were obtained by culturing Hepa1-6 cells in DMSO-DMEM which containing 2% vol DMSO (Sigma, USA) in DMEM supplemented with 10% FBS and 1% 100 U/ml penicillin-streptomycin for 7 days before performing experiments.

C57BL/6 mice, nude mice and NOD/SCID mice were purchased from Shanghai Research Center for Model Organisms, and raised in specific pathogen-free (SPF) animal rooms of Department of Cell Biology, SMMU. This study was approved by the Institutional Animal Care and Use Committee of Second Military Medical University.

### Cell proliferation, cell cycle and cell viability assay

A cell count kit-8 (CCK-8) (Dojindo, Japan) was used to examine cell proliferation and cell viability. Hep and D-hep cells were seeded into 96-well plates at 1 × 10^3^ cells per well in complete DMEM medium with or without 2% DMSO. The day of seeding is day 0 and the medium of D-hep group was changed into DMSO-medium at day 1. Following the manufacturer's instructions, on day 0 and day 1, 2, 3, 4, 5, 6 and 7, medium was removed and 100 ml DMEM containing CCK-8 (10%) was added to each well. After 2 h incubation at 37°C, the absorbance at 450 nm of each well was measured using a Microtiter Plate Reader (TECAN, Switzerland). The average absorbance of 10 independent wells for each group were obtained. The proliferating rate everyday was presented by the ratio of absorbance value of day 1∼7 to value of day 0.

Cell cycle for D-hep cells, Hep cells and Df cells, D-hep cells cultured in DMSO-free medium for 7 days, was analyzed by using DNA staining Kit (BD, USA) and subjected to FACS LSR II (BD, USA) for flow cytometry. 1 × 10^6^ cells in each sample and three independent wells were analyzed.

Cell viability for Hep cells cultured in DMSO-medium for 1 day (DM-1) and 7 days (D-hep) were analyzed by seeding 1 × 10^5^, 1 × 10^4^, 1 × 10^3^ cells of each type in 96-well plate and using CCK-8 reagent to monitor cell viability. Three independent wells were analyzed.

### Colony forming efficiency (CFE) assay

Hep cells were seeded into 6-well plates at 1 × 10^3^ cells per well and cultured in complete DMEM culture medium with or without 2% DMSO. 14 days later, cells were fixed with 70% ethanol and stained with toluidine blue O (Sigma, USA). Clones (diameter of more than 0.5 mm) were counted and the results were shown as the average number of clones counted in 6 independent wells.

### Detection of apoptosis and necrosis

Apoptotic cells and necrotic cells were analyzed by double staining with Alexa Fluor 488 annexin V and propidium iodide (PI) (BD, USA). All cells at exponential phase, including floating and adherent cells, were collected and washed for three times with 4°C PBS. 5 μl Alexa Fluor 488 Annexin V was added to the cell suspension in the presence of 195 μl binding buffer and incubated for 20 min at room temperature. Cells were co-stained with 5 μl PI and immediately analyzed using FACS LSR II (BD, USA). The percentage of apoptotic (annexinV) and necrotic (PI) cells was determined using software. Data represent the mean fluorescence obtained from a population of 10,000 cells.

### Establishment of a D-hep-C57 mouse model with anti-tumor immunity against Hepa1-6 cells

1 × 10^6^ D-hep cells were injected subcutaneously into the inguen of 8- to 10-week-old C57BL/6 mice. After tumor formation and regression, the mice that developed anti-tumor immunity against Hepa1-6 cells were regarded as D-hep-C57 mice.

### Tumorigenicity *in vivo*

Briefly, 1 × 10^6^ D-hep cells or Hep cells were injected subcutaneously into the inguen of C57BL/6 (3 mice per group), NOD/SCID mice (6 mice per group) and nude mice (3 mice per group). The C57BL/6 mice were sacrificed 1, 2, 3 and 4 weeks after injection, NOD/SCID mice were sacrificed 4 weeks after the injection and nude mice were sacrificed 20 or 30 days after the injection. Tumors were harvested and weighed. The results are shown as the average weights of the tumors.

Besides, to confirm the therapeutic effect of D-hep cells inducing antitumor immunity, 1 × 10^6^ D-hep cells were injected subcutaneously into one side of inguen of 8- to 10-week-old C57BL/6 mice while 1 × 10^6^ of Hep cells were injected into the other side. Tumor size was calculated with nonius each 10 days after cell injection. The tumor volume was calculated as (length *width^2^)/2 or 4πR^3^/3 (R is tumor radius) depending on the tumor shape. Tumor size was shown as the average of 6 tumors per group.

To evaluate the safety of D-hep cells, 1 × 10^7^ D-hep cells were injected into the inguen of C57BL/6 mice as above described with 1 × 10^7^ Hep cells as control. Tumor size was shown as the average of 6 tumors per group.

### Flow cytometry analysis

The following antibodies were purchased: Anti-Mouse CD62L (L-Selectin)-FITC, Anti-Mouse-NK1.1-FITC, Anti-Mouse-CD44-PE (eBioscience, USA), Anti-Mouse-CD4-APC and Anti-Mouse-CD8a-APC (R & D Systems, USA), Anti-Mouse-CD25-FITC and Anti-Mouse-Foxp3-PE (BD, USA). The Foxp3-Transcription Factor Staining Buffer Set was purchased from eBioscience (USA). Cells were stained following the manufacturers' protocols with optimal concentrations of mAb. Briefly, splenocytes from individual mice were prepared in PBS and stained with antibodies at 4°C for 15 minutes. After washing three times, the cells were resuspended in washing buffer and analyzed. Fluorescent data for 10,000 lymphocyte events each sample were acquired on a FACS LSR II (BD Bioscience, USA) and were analyzed using FlowJo software (Tomy Digital Biology Co., Ltd., Japan).

### Enzyme-linked immunosorbent assays (ELISAs) of cytokine

Mouse blood was harvested from the postocular venous plexus and centrifuged. The cytokine level in the mouse serum was detected using IFN-γ or IL-2 ELISA kits (Excell, China) following the manufacturer's instructions and were measured using Microtiter Plate Reader (TECAN, Switzerland) at 450 nm. At least three mice per group were included in the analysis.

### Immunohistochemistry assay of tumor infiltrating lymphocytes (TILs)

Immunohistochemistry was performed on formalin-fixed paraffin-embedded tumor tissues of 7, 14, 21 and 28 days after the injection of D-hep or Hep cells. Briefly, after being deparaffinized, rehydrated and antigen retrieval with tris-EDTA buffer (pH = 9.0), 3 um-thick slides were blocked with 1% BSA in PBS for 1 h at room temperature. The slides were then incubated with the primary CD4 and CD8 monoclonal antibody (BD, USA) at the appropriate dilution at 4°C overnight and then washed by PBST (PBS with 0.2% Tween-20, pH = 7.1) for 3 times. Secondary antibody was incubated at 37°C for 30 min and then slides were washed by PBST for 3 times. After visualized by DAB substrate and hematoxylin counterstain, slides were mounted for microscopy.

### Cytotoxicity assays

According to the method proposed by Silaeva YY *et al.* [27], splenocytes were isolated and co-cultured with Hepa1-6 cells in 96-well, U-bottom plates at a ratio of 50 effector cells (50,000 cells) to 1 target cell (1,000 cells) or 100 effector cells (100,000 cells) to 1 target cell (1,000 cells). The plates were then briefly centrifuged and cultured at 37°C for 3, 4 or 5 days in complete DMEM. After collecting the splenocytes for the antigen-specific T cell proliferation assays, the wells were washed 5 times to remove the residual splenocytes and then CCK-8 analysis was performed to test the viability of tumor cells

### *In vitro* splenic T cell proliferation assays

According to the method proposed by Silaeva YY *et al.* [27], mouse splenocytes were suspended in 0.1% FBS/PBS at a density of 2 × 10^7^/ml and then pre-labelled with 5 μM carboxyfluorescein succinimidyl ester (CFSE, Sigma, USA) for 10 minutes at 37°C. Isovolumetric 100% FBS was then added, and the splenocytes were incubated in a 37°C water bath for 10 minutes. After the incubation, the splenocytes were centrifuged (400 g, 5 minutes) and washed three times with 2% FBS/PBS wash buffer (1 ml for 10^6^ splenocytes). After washing, the splenocytes were co-cultured with Hepa1-6 cells for 3 days, and then were collected, stained with CD4 and CD8 antibodies, subjected to flow cytometry and detected.

### Statistical analysis

The data were analyzed by SPSS 16.0 software. Student's *t*-test or non-parametric test was used for comparison, and *P* values < 0.05 or < 0.01 were considered statistically significant. The data are shown as the means ± standard deviations (S.D.).

## SUPPLEMENTARY MATERIALS FIGURES AND TABLE




