# Supplementary material for: Altered Hepa1-6 cells by dimethyl sulfoxide (DMSO)-treatment induce anti-tumor immunity in vivo
Source: Oncotarget. 2016 Jan 25;7(8):9340–52. doi: 10.18632/oncotarget.7009 (PMC4891044; doi:10.18632/oncotarget.7009)
Supplement: Supplementary file 1 [file oncotarget-07-9340-s001.pdf]

Altered Hepa1-6 cells by dimethyl sulfoxide (DMSO)-treatment induce anti-tumor immunity *in vivo*

Supplementary Materials

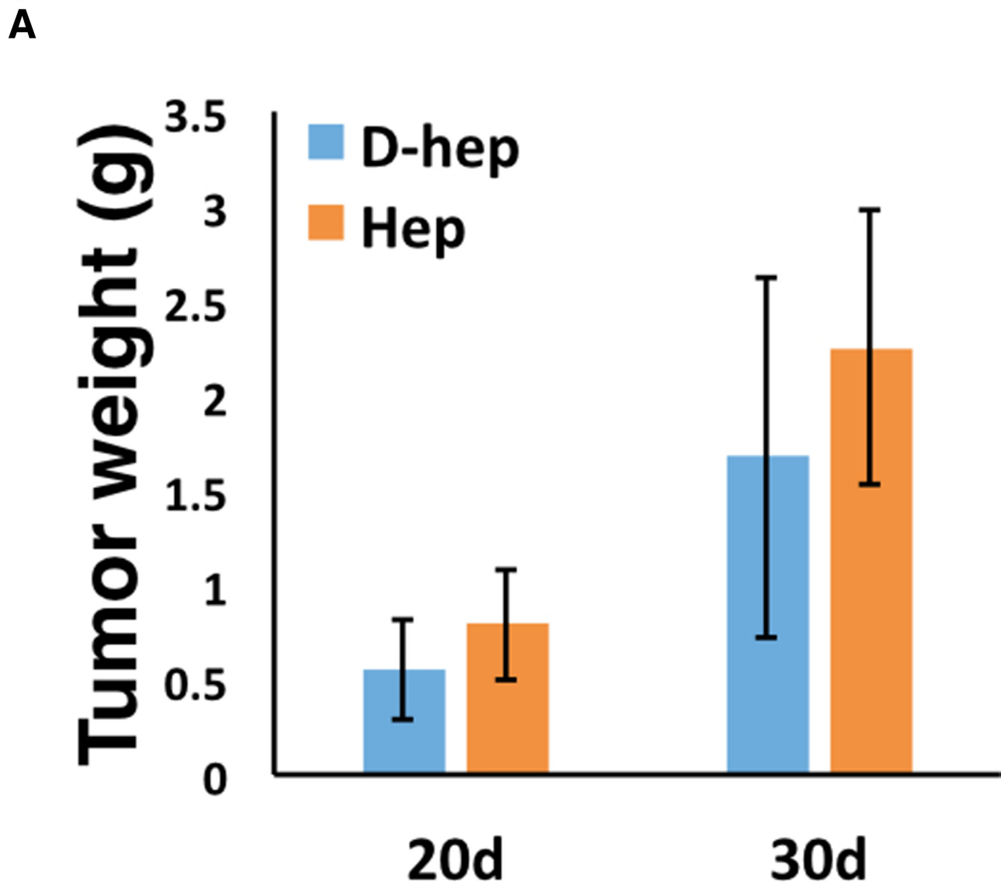

Supplementary Figure S1: Tumorigenicity of Hep or D-hep cells in nude mice. (**A**) D-hep tumors and Hep tumors could form and grow successfully *in vivo* 20 or 30 days after the injection ($n = 3$). The error bars represent ± S.D.; n = biological replicates.

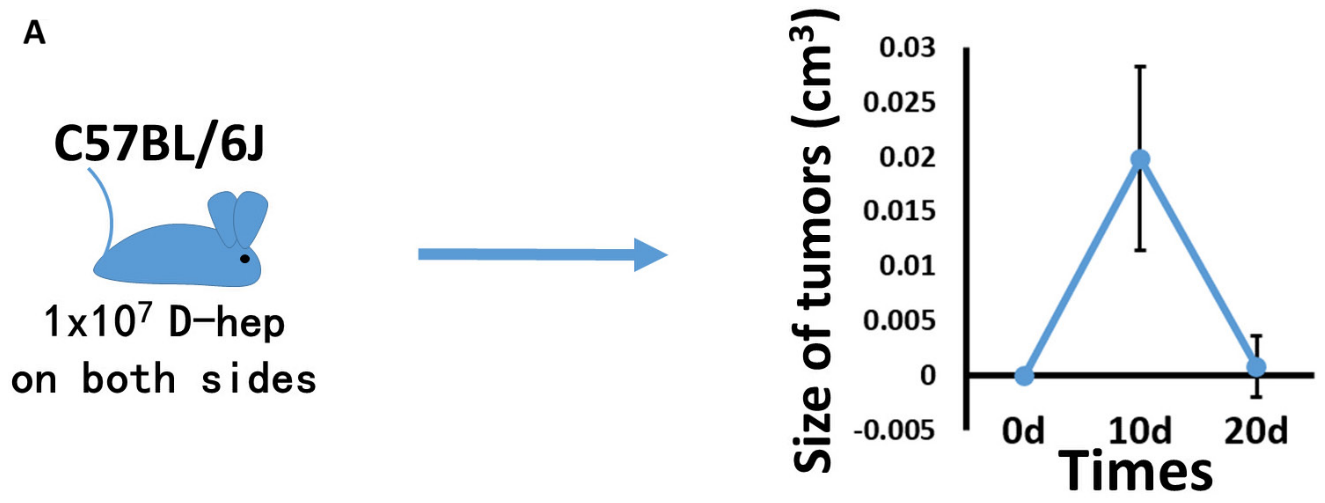

Supplementary Figure S2: Tumorigenicity of 1 × 10^7 D-hep cells in C57BL/6 mice. (**A**) D-hep tumors were formed and regressed in 20 days after the injection (*n* = 6). The error bars represent ± S.D.; *n* = biological replicates.

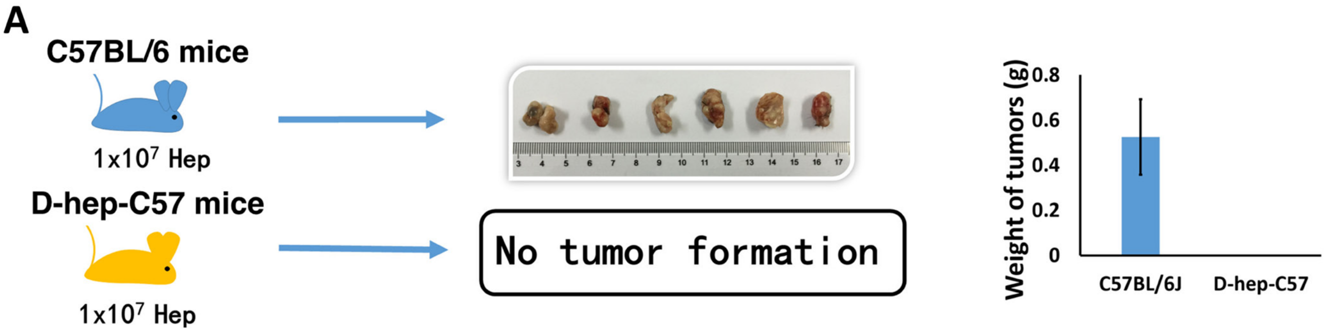

Supplementary Figure S3: Tumorigenicity of 1 × 10^7 Hep cells in D-hep-C57 and WT-C57 mice. (**A**) No tumor formation in D-hep-C57 mice were observed while tumors in WT-C57 mice kept growing (*n* = 6). The error bars represent ± S.D.; *n* = biological replicates.

A

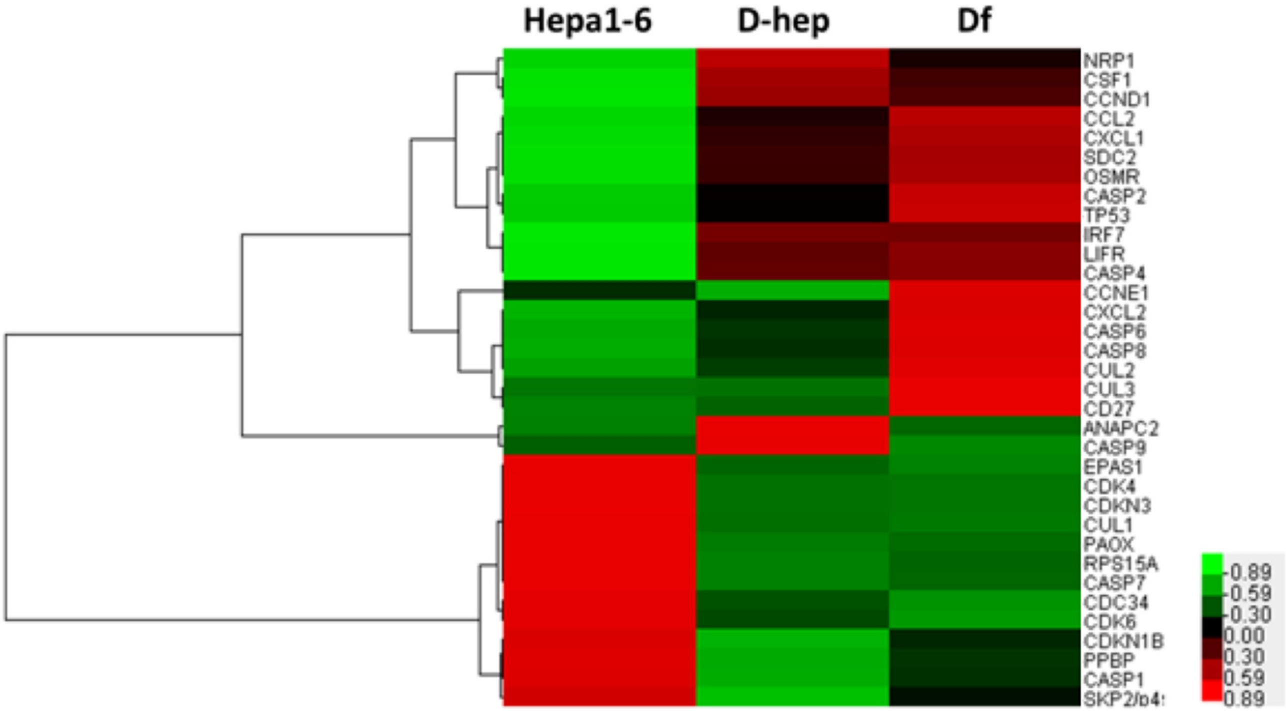

Supplementary Figure 4: Heatmap from quantitative PCR array of selected genes in D-hep, Hep and Df cells.

Supplementary Table S1: Kyoto Encyclopedia of Genes and Genomes (KEGG) analysis of altered genes in D-hep cells compared with Hep cells.
