# Supplementary material for: Altered Hepa1-6 cells by dimethyl sulfoxide (DMSO)-treatment induce anti-tumor immunity in vivo
Source: Oncotarget. 2016 Jan 25;7(8):9340–52. doi: 10.18632/oncotarget.7009 (PMC4891044; doi:10.18632/oncotarget.7009)
Supplement: Supplementary file 2 [file oncotarget-07-9340-s002.docx]

|  |  | **log2 (Ratio)**  **D-hep/Hep** | **P-value**  **(Differentially expressed)**  **D-hep/Hep** |
| --- | --- | --- | --- |
| **KEGG_RIBOSOME** |  |  |  |
| FAU | Finkel-Biskis-Reilly murine sarcoma virus (FBR-MuSV) ubiquitously expressed (fox derived); ribosomal protein S30 | -1.402714045 | 0.003773927 |
| RPL10A | ribosomal protein L10a | -1.816834066 | 0.00215027 |
| RPL14 | ribosomal protein L14 | -1.265828291 | 0.009586746 |
| RPL24 | ribosomal protein L24 | -1.09181918 | 0.026770415 |
| RPL27A | ribosomal protein L27a | -0.890751958 | 0.044540037 |
| RPL30 | ribosomal protein L30 | -0.903212939 | 0.041656666 |
| RPL34 | ribosomal protein L34 | -0.982473376 | 0.034359619 |
| RPL38 | ribosomal protein L38 | -0.884394003 | 0.046577819 |
| RPLP0 | ribosomal protein, large, P0 | -1.430104897 | 0.00345247 |
| RPS15A | ribosomal protein S15a | -2.061667727 | 0.000327058 |
| RPS24 | ribosomal protein S24 | -1.769116888 | 0.000775464 |
| RPS25 | ribosomal protein S25 | -1.544963086 | 0.003409581 |
| RPS3 | ribosomal protein S3 | -1.108100023 | 0.026487416 |
| RPS7 | ribosomal protein S7 | -1.135892718 | 0.035539474 |
| **KEGG_LYSOSOME** |  |  |  |
| GLB1 | galactosidase, beta 1 | 0.924549075 | 0.038631767 |
| HEXB | hexosaminidase B (beta polypeptide) | 0.971268027 | 0.032155458 |
| ARSB | arylsulfatase B | 1.046950761 | 0.025732709 |
| HGSNAT | heparan-alpha-glucosaminide N-acetyltransferase | 1.634847341 | 0.001326987 |
| SGSH | N-sulfoglucosamine sulfohydrolase (sulfamidase) | 0.985672299 | 0.036779661 |
| MAN2B1 | mannosidase, alpha, class 2B, member 1 | 1.121448945 | 0.016019693 |
| SORT1 | sortilin 1 | 1.380701136 | 0.004838616 |
| LIPA | lipase A, lysosomal acid, cholesterol esterase (Wolman disease) | 1.538265756 | 0.002967344 |
| AP1G1 | adaptor-related protein complex 1, gamma 1 subunit | 1.001244492 | 0.036597487 |
| AP3M2 | adaptor-related protein complex 3, mu 2 subunit | 0.983968492 | 0.040134985 |
| CLN3 | ceroid-lipofuscinosis, neuronal 3, juvenile (Batten, Spielmeyer-Vogt disease) | -1.102004122 | 0.019186009 |
| CLN5 | ceroid-lipofuscinosis, neuronal 5 | 1.474819371 | 0.006490792 |
| GNPTAB | N-acetylglucosamine-1-phosphate transferase, alpha and beta subunits | 1.192269037 | 0.034154575 |
| LAMP2 | lysosomal-associated membrane protein 2 | 1.165141428 | 0.011948752 |
| SCARB2 | scavenger receptor class B, member 2 | 1.346733634 | 0.028295163 |
| SUMF1 | sulfatase modifying factor 1 | 1.386438946 | 0.017583484 |
| **KEGG_CYTOKINE_CYTOKINE_RECEPTOR_INTERACTION** |  |  |  |
| CCL5 | chemokine (C-C motif) ligand 5 | NA | NA |
| VEGFB | vascular endothelial growth factor B | -1.250197889 | 0.008209675 |
| VEGFC | vascular endothelial growth factor C | -1.717939619 | 0.001511676 |
| TGFBR2 | transforming growth factor, beta receptor II (70/80kDa) | 0.88481149 | 0.045336518 |
| CXCL1 | chemokine (C-X-C motif) ligand 1 (melanoma growth stimulating activity, alpha) | 3.515131665 | 7.46425E-06 |
| CCL2 | chemokine (C-C motif) ligand 2 | 3.645861129 | 5.10199E-06 |
| CXCL2 | chemokine (C-X-C motif) ligand 2 | 3.865186349 | 5.04803E-06 |
| CCL17 | chemokine (C-C motif) ligand 17 | 1.540563474 | 0.006678958 |
| CX3CL1 | chemokine (C-X3-C motif) ligand 1 | 1.03477223 | 0.022292476 |
| CXCL14 | chemokine (C-X-C motif) ligand 14 | -1.223470343 | 0.014916104 |
| CXCL5 | chemokine (C-X-C motif) ligand 5 | 1.490463437 | 0.012881711 |
| PPBP | pro-platelet basic protein (chemokine (C-X-C motif) ligand 7) | -3.258112534 | 3.13353E-05 |
| LIF | leukemia inhibitory factor (cholinergic differentiation factor) | 1.536203464 | 0.003559406 |
| CSF2 | colony stimulating factor 2 (granulocyte-macrophage) | NA | NA |
| IL6ST | interleukin 6 signal transducer (gp130, oncostatin M receptor) | 1.104173733 | 0.020177528 |
| IL20RB | interleukin 20 receptor beta | 1.034051697 | 0.023009289 |
| LIFR | leukemia inhibitory factor receptor alpha | 2.422574124 | 0.000226366 |
| OSMR | oncostatin M receptor | 2.317583381 | 0.000543184 |
| CSF1 | colony stimulating factor 1 (macrophage) | 2.008134032 | 0.000324265 |
| BMPR2 | bone morphogenetic protein receptor, type II (serine/threonine kinase) | 1.373922227 | 0.013060817 |
| LTBR | lymphotoxin beta receptor (TNFR superfamily, member 3) | 1.071527009 | 0.020935657 |
| EDA2R | ectodysplasin A2 receptor | 1.341723087 | 0.005360754 |
| IL17RA | interleukin 17 receptor A | 0.937903741 | 0.04447446 |
| **KEGG_TOLL_LIKE_RECEPTOR_SIGNALING_PATHWAY** |  |  |  |
| CCL5 | chemokine (C-C motif) ligand 5 | NA | NA |
| NFKBIA | nuclear factor of kappa light polypeptide gene enhancer in B-cells inhibitor, alpha | 1.230827121 | 0.008576939 |
| PIK3R1 | phosphoinositide-3-kinase, regulatory subunit 1 (p85 alpha) | 0.999787095 | 0.042866007 |
| PIK3R3 | phosphoinositide-3-kinase, regulatory subunit 3 (p55, gamma) | 1.006628968 | 0.048930194 |
| FOS | v-fos FBJ murine osteosarcoma viral oncogene homolog | 1.532413004 | 0.002073657 |
| CD14 | CD14 molecule | -1.477953747 | 0.003993452 |
| MAP2K4 | mitogen-activated protein kinase kinase 4 | 1.011406469 | 0.031394709 |
| MAP2K6 | mitogen-activated protein kinase kinase 6 | -0.976346687 | 0.046764851 |
| TLR2 | toll-like receptor 2 | 1.154309315 | 0.043522142 |
| SPP1 | secreted phosphoprotein 1 (osteopontin, bone sialoprotein I, early T-lymphocyte activation 1) | -1.747361776 | 0.000845446 |
| LBP | lipopolysaccharide binding protein | 1.530094003 | 0.002702839 |
| IRF7 | interferon regulatory factor 7 | 2.253938298 | 0.000236444 |
| IRF5 | interferon regulatory factor 5 | -1.317265541 | 0.014099669 |
| **KEGG_BLADDER_CANCER** |  |  |  |
| VEGFB | vascular endothelial growth factor B | -1.250197889 | 0.008209675 |
| VEGFC | vascular endothelial growth factor C | -1.717939619 | 0.001511676 |
| MDM2 | Mdm2, transformed 3T3 cell double minute 2, p53 binding protein (mouse) | 0.973982046 | 0.030622464 |
| PGF | placental growth factor, vascular endothelial growth factor-related protein | -1.387482912 | 0.014012369 |
| CDKN2A | cyclin-dependent kinase inhibitor 2A (melanoma, p16, inhibits CDK4) | -1.505542503 | 0.003536159 |
| RASSF1 | Ras association (RalGDS/AF-6) domain family 1 | -1.193082232 | 0.012603768 |
| DAPK3 | death-associated protein kinase 3 | -1.018503466 | 0.029363997 |
| RPS6KA5 | ribosomal protein S6 kinase, 90kDa, polypeptide 5 | 0.93686799 | 0.041860294 |
| **KEGG_PATHWAYS_IN_CANCER** |  |  |  |
| VEGFB | vascular endothelial growth factor B | -1.250197889 | 0.008209675 |
| VEGFC | vascular endothelial growth factor C | -1.717939619 | 0.001511676 |
| TGFBR2 | transforming growth factor, beta receptor II (70/80kDa) | 0.88481149 | 0.045336518 |
| NFKBIA | nuclear factor of kappa light polypeptide gene enhancer in B-cells inhibitor, alpha | 1.230827121 | 0.008576939 |
| PIK3R1 | phosphoinositide-3-kinase, regulatory subunit 1 (p85 alpha) | 0.999787095 | 0.042866007 |
| PIK3R3 | phosphoinositide-3-kinase, regulatory subunit 3 (p55, gamma) | 1.006628968 | 0.048930194 |
| FOS | v-fos FBJ murine osteosarcoma viral oncogene homolog | 1.532413004 | 0.002073657 |
| MDM2 | Mdm2, transformed 3T3 cell double minute 2, p53 binding protein (mouse) | 0.973982046 | 0.030622464 |
| PGF | placental growth factor, vascular endothelial growth factor-related protein | -1.387482912 | 0.014012369 |
| CDKN2A | cyclin-dependent kinase inhibitor 2A (melanoma, p16, inhibits CDK4) | -1.505542503 | 0.003536159 |
| RASSF1 | Ras association (RalGDS/AF-6) domain family 1 | -1.193082232 | 0.012603768 |
| DAPK3 | death-associated protein kinase 3 | -1.018503466 | 0.029363997 |
| BIRC2 | baculoviral IAP repeat-containing 2 | 1.137727113 | 0.016708782 |
| RBX1 | ring-box 1 | -0.894936836 | 0.045340549 |
| PML | promyelocytic leukemia | 1.081700567 | 0.032688517 |
| FGFR2 | fibroblast growth factor receptor 2 (bacteria-expressed kinase, keratinocyte growth factor receptor, craniofacial dysostosis 1, Crouzon syndrome, Pfeiffer syndrome, Jackson-Weiss syndrome) | 1.414670515 | 0.007769214 |
| ITGB1 | integrin, beta 1 (fibronectin receptor, beta polypeptide, antigen CD29 includes MDF2, MSK12) | 0.904558067 | 0.0411008 |
| ITGAV | integrin, alpha V (vitronectin receptor, alpha polypeptide, antigen CD51) | 1.19035653 | 0.01082119 |
| LAMB1 | laminin, beta 1 | 0.992639608 | 0.031675194 |
| EPAS1 | endothelial PAS domain protein 1 | 2.4268684 | 8.157E-05 |
| MSH6 | mutS homolog 6 (E. coli) | 1.773238108 | 0.002221183 |
| **KEGG_GLYCINE_SERINE_AND_THREONINE_METABOLISM** |  |  |  |
| DLD | dihydrolipoamide dehydrogenase | 1.385239943 | 0.007644305 |
| SHMT1 | serine hydroxymethyltransferase 1 (soluble) | -1.34615804 | 0.005403182 |
| SHMT2 | serine hydroxymethyltransferase 2 (mitochondrial) | -1.359824559 | 0.004732953 |
| CTH | cystathionase (cystathionine gamma-lyase) | -1.488807742 | 0.009917175 |
| GCAT | glycine C-acetyltransferase (2-amino-3-ketobutyrate coenzyme A ligase) | -1.342025635 | 0.005547871 |
| PSPH | phosphoserine phosphatase | -0.996052375 | 0.028478634 |
| SARDH | sarcosine dehydrogenase | 1.413955825 | 0.006727203 |
| **KEGG_GLYCOSAMINOGLYCAN_DEGRADATION** |  |  |  |
| GLB1 | galactosidase, beta 1 | 0.924549075 | 0.038631767 |
| HEXB | hexosaminidase B (beta polypeptide) | 0.971268027 | 0.032155458 |
| ARSB | arylsulfatase B | 1.046950761 | 0.025732709 |
| HGSNAT | heparan-alpha-glucosaminide N-acetyltransferase | 1.634847341 | 0.001326987 |
| SGSH | N-sulfoglucosamine sulfohydrolase (sulfamidase) | 0.985672299 | 0.036779661 |
| HS3ST3B1 | heparan sulfate (glucosamine) 3-O-sulfotransferase 3B1 | 1.432523002 | 0.006707726 |
| **KEGG_CHEMOKINE_SIGNALING_PATHWAY** |  |  |  |
| CCL5 | chemokine (C-C motif) ligand 5 | NA | NA |
| CXCL1 | chemokine (C-X-C motif) ligand 1 (melanoma growth stimulating activity, alpha) | 3.515131665 | 7.46425E-06 |
| CCL2 | chemokine (C-C motif) ligand 2 | 3.645861129 | 5.10199E-06 |
| CXCL2 | chemokine (C-X-C motif) ligand 2 | 3.865186349 | 5.04803E-06 |
| CCL17 | chemokine (C-C motif) ligand 17 | 1.540563474 | 0.006678958 |
| CX3CL1 | chemokine (C-X3-C motif) ligand 1 | 1.03477223 | 0.022292476 |
| CXCL14 | chemokine (C-X-C motif) ligand 14 | -1.223470343 | 0.014916104 |
| CXCL5 | chemokine (C-X-C motif) ligand 5 | 1.490463437 | 0.012881711 |
| PPBP | pro-platelet basic protein (chemokine (C-X-C motif) ligand 7) | -3.258112534 | 3.13353E-05 |
| NFKBIA | nuclear factor of kappa light polypeptide gene enhancer in B-cells inhibitor, alpha | 1.230827121 | 0.008576939 |
| PIK3R1 | phosphoinositide-3-kinase, regulatory subunit 1 (p85 alpha) | 0.999787095 | 0.042866007 |
| PIK3R3 | phosphoinositide-3-kinase, regulatory subunit 3 (p55, gamma) | 1.006628968 | 0.048930194 |
| ARRB1 | arrestin, beta 1 | 1.040593202 | 0.027286537 |
| STAT2 | signal transducer and activator of transcription 2, 113kDa | 1.299573041 | 0.026090128 |
| GNG10 | guanine nucleotide binding protein (G protein), gamma 10 | -1.493305363 | 0.00372498 |
| **BIOCARTA_TOLL_PATHWAY** |  |  |  |
| NFKBIA | nuclear factor of kappa light polypeptide gene enhancer in B-cells inhibitor, alpha | 1.230827121 | 0.008576939 |
| FOS | v-fos FBJ murine osteosarcoma viral oncogene homolog | 1.532413004 | 0.002073657 |
| CD14 | CD14 molecule | -1.477953747 | 0.003993452 |
| MAP2K4 | mitogen-activated protein kinase kinase 4 | 1.011406469 | 0.031394709 |
| MAP2K6 | mitogen-activated protein kinase kinase 6 | -0.976346687 | 0.046764851 |
| TLR2 | toll-like receptor 2 | 1.154309315 | 0.043522142 |
| EIF2AK2 | eukaryotic translation initiation factor 2-alpha kinase 2 | 1.141346002 | 0.035182513 |
| **KEGG_PURINE_METABOLISM** |  |  |  |
| POLE4 | polymerase (DNA-directed), epsilon 4 (p12 subunit) | 1.012767041 | 0.034025561 |
| POLR1B | polymerase (RNA) I polypeptide B, 128kDa | -1.135878759 | 0.016378481 |
| POLR2H | polymerase (RNA) II (DNA directed) polypeptide H | -0.944306445 | 0.034725364 |
| PRIM1 | primase, polypeptide 1, 49kDa | 1.03032361 | 0.026531892 |
| NME4 | non-metastatic cells 4, protein expressed in | -1.298103367 | 0.00691251 |
| NME6 | non-metastatic cells 6, protein expressed in (nucleoside-diphosphate kinase) | -1.518539181 | 0.002669182 |
| PDE2A | phosphodiesterase 2A, cGMP-stimulated | -1.467832164 | 0.00364542 |
| PRPS2 | phosphoribosyl pyrophosphate synthetase 2 | 1.078549359 | 0.044146139 |
| AK1 | adenylate kinase 1 | -1.484447532 | 0.006197008 |
| AK2 | adenylate kinase 2 | -1.077429853 | 0.023944387 |
| AMPD3 | adenosine monophosphate deaminase (isoform E) | -1.473373755 | 0.003743769 |
| PFAS | phosphoribosylformylglycinamidine synthase (FGAR amidotransferase) | -0.893229462 | 0.044022683 |
| PRUNE | prune homolog (Drosophila) | -1.081890747 | 0.042307902 |
| **KEGG_UBIQUITIN_MEDIATED_PROTEOLYSIS** |  |  |  |
| MDM2 | Mdm2, transformed 3T3 cell double minute 2, p53 binding protein (mouse) | 0.973982046 | 0.030622464 |
| BIRC2 | baculoviral IAP repeat-containing 2 | 1.137727113 | 0.016708782 |
| RBX1 | ring-box 1 | -0.894936836 | 0.045340549 |
| PML | promyelocytic leukemia | 1.081700567 | 0.032688517 |
| SOCS3 | suppressor of cytokine signaling 3 | 1.644504309 | 0.001312385 |
| CDC27 | cell division cycle 27 | 1.637207727 | 0.005239304 |
| CUL4B | cullin 4B | 1.059755076 | 0.037360698 |
| UBE2A | ubiquitin-conjugating enzyme E2A (RAD6 homolog) | 0.963579398 | 0.047747519 |
| UBE2G2 | ubiquitin-conjugating enzyme E2G 2 (UBC7 homolog, yeast) | -1.072767098 | 0.033386927 |
| DET1 | de-etiolated homolog 1 (Arabidopsis) | -1.254155657 | 0.010051943 |
| UBE2B | ubiquitin-conjugating enzyme E2B (RAD6 homolog) | 1.709633616 | 0.004436221 |
| UBE2C | ubiquitin-conjugating enzyme E2C | -0.901484971 | 0.04448355 |
| **KEGG_MAPK_SIGNALING_PATHWAY** |  |  |  |
| TGFBR2 | transforming growth factor, beta receptor II (70/80kDa) | 0.88481149 | 0.045336518 |
| FOS | v-fos FBJ murine osteosarcoma viral oncogene homolog | 1.532413004 | 0.002073657 |
| CD14 | CD14 molecule | -1.477953747 | 0.003993452 |
| MAP2K4 | mitogen-activated protein kinase kinase 4 | 1.011406469 | 0.031394709 |
| MAP2K6 | mitogen-activated protein kinase kinase 6 | -0.976346687 | 0.046764851 |
| RPS6KA5 | ribosomal protein S6 kinase, 90kDa, polypeptide 5 | 0.93686799 | 0.041860294 |
| FGFR2 | fibroblast growth factor receptor 2 (bacteria-expressed kinase, keratinocyte growth factor receptor, craniofacial dysostosis 1, Crouzon syndrome, Pfeiffer syndrome, Jackson-Weiss syndrome) | 1.414670515 | 0.007769214 |
| ARRB1 | arrestin, beta 1 | 1.040593202 | 0.027286537 |
| FGFR4 | fibroblast growth factor receptor 4 | -1.539435158 | 0.003358729 |
| MRAS | muscle RAS oncogene homolog | -1.70801693 | 0.001280061 |
| NFATC4 | nuclear factor of activated T-cells, cytoplasmic, calcineurin-dependent 4 | 1.112630511 | 0.046243876 |
| RASGRP3 | RAS guanyl releasing protein 3 (calcium and DAG-regulated) | -1.732132427 | 0.001917594 |
| GADD45B | growth arrest and DNA-damage-inducible, beta | 1.464315445 | 0.003277579 |
| CACNB3 | calcium channel, voltage-dependent, beta 3 subunit | -0.948256393 | 0.039457046 |
| MAP4K1 | mitogen-activated protein kinase kinase kinase kinase 1 | -1.387482912 | 0.014011849 |
| MAPKAPK3 | mitogen-activated protein kinase-activated protein kinase 3 | -0.945078454 | 0.039870132 |
| DUSP5 | dual specificity phosphatase 5 | 1.161662725 | 0.026617928 |
| **KEGG_REGULATION_OF_ACTIN_CYTOSKELETON** |  |  |  |
| PIK3R1 | phosphoinositide-3-kinase, regulatory subunit 1 (p85 alpha) | 0.999787095 | 0.042866007 |
| PIK3R3 | phosphoinositide-3-kinase, regulatory subunit 3 (p55, gamma) | 1.006628968 | 0.048930194 |
| CD14 | CD14 molecule | -1.477953747 | 0.003993452 |
| FGFR2 | fibroblast growth factor receptor 2 (bacteria-expressed kinase, keratinocyte growth factor receptor, craniofacial dysostosis 1, Crouzon syndrome, Pfeiffer syndrome, Jackson-Weiss syndrome) | 1.414670515 | 0.007769214 |
| ITGB1 | integrin, beta 1 (fibronectin receptor, beta polypeptide, antigen CD29 includes MDF2, MSK12) | 0.904558067 | 0.0411008 |
| ITGAV | integrin, alpha V (vitronectin receptor, alpha polypeptide, antigen CD51) | 1.19035653 | 0.01082119 |
| FGFR4 | fibroblast growth factor receptor 4 | -1.539435158 | 0.003358729 |
| MRAS | muscle RAS oncogene homolog | -1.70801693 | 0.001280061 |
| PAK6 | p21(CDKN1A)-activated kinase 6 | -1.282458411 | 0.010937677 |
| VCL | vinculin | 1.261421551 | 0.008152852 |
| ARHGEF12 | Rho guanine nucleotide exchange factor (GEF) 12 | 1.045940749 | 0.024952229 |
| GSN | gelsolin (amyloidosis, Finnish type) | -1.175444182 | 0.013341403 |
| CHRM5 | cholinergic receptor, muscarinic 5 | -1.073040141 | 0.026693473 |
| IQGAP2 | IQ motif containing GTPase activating protein 2 | 1.130681992 | 0.034675982 |
| RDX | radixin | 1.058125913 | 0.031967949 |
| **KEGG_ABC_TRANSPORTERS** |  |  |  |
| ABCD3 | ATP-binding cassette, sub-family D (ALD), member 3 | 0.974323895 | 0.038760725 |
| ABCC3 | ATP-binding cassette, sub-family C (CFTR/MRP), member 3 | -1.162204657 | 0.014985315 |
| ABCA1 | ATP-binding cassette, sub-family A (ABC1), member 1 | 1.48693543 | 0.003247678 |
| ABCB10 | ATP-binding cassette, sub-family B (MDR/TAP), member 10 | 1.287058797 | 0.019630874 |
| ABCB7 | ATP-binding cassette, sub-family B (MDR/TAP), member 7 | 0.928123712 | 0.046631146 |
| ABCG1 | ATP-binding cassette, sub-family G (WHITE), member 1 | 1.12476578 | 0.04442912 |
| ABCG2 | ATP-binding cassette, sub-family G (WHITE), member 2 | 1.642827925 | 0.00143455 |
| **KEGG_NOD_LIKE_RECEPTOR_SIGNALING_PATHWAY** |  |  |  |
| CCL5 | chemokine (C-C motif) ligand 5 | NA | NA |
| CXCL1 | chemokine (C-X-C motif) ligand 1 (melanoma growth stimulating activity, alpha) | 3.515131665 | 7.46425E-06 |
| CCL2 | chemokine (C-C motif) ligand 2 | 3.645861129 | 5.10199E-06 |
| CXCL2 | chemokine (C-X-C motif) ligand 2 | 3.865186349 | 5.04803E-06 |
| NFKBIA | nuclear factor of kappa light polypeptide gene enhancer in B-cells inhibitor, alpha | 1.230827121 | 0.008576939 |
| BIRC2 | baculoviral IAP repeat-containing 2 | 1.137727113 | 0.016708782 |
| CASP1 | caspase 1, apoptosis-related cysteine peptidase (interleukin 1, beta, convertase) | 2.223619585 | 0.000292933 |
| TNFAIP3 | tumor necrosis factor, alpha-induced protein 3 | 1.91939532 | 0.000540621 |
| **BIOCARTA_RNA_PATHWAY** |  |  |  |
| NFKBIA | nuclear factor of kappa light polypeptide gene enhancer in B-cells inhibitor, alpha | 1.230827121 | 0.008576939 |
| EIF2AK2 | eukaryotic translation initiation factor 2-alpha kinase 2 | 1.141346002 | 0.035182513 |
| EIF2S2 | eukaryotic translation initiation factor 2, subunit 2 beta, 38kDa | -1.202444475 | 0.021486018 |
| DNAJC3 | DnaJ (Hsp40) homolog, subfamily C, member 3 | 1.72522868 | 0.004005216 |
| **KEGG_FOCAL_ADHESION** |  |  |  |
| VEGFB | vascular endothelial growth factor B | -1.250197889 | 0.008209675 |
| VEGFC | vascular endothelial growth factor C | -1.717939619 | 0.001511676 |
| PIK3R1 | phosphoinositide-3-kinase, regulatory subunit 1 (p85 alpha) | 0.999787095 | 0.042866007 |
| PIK3R3 | phosphoinositide-3-kinase, regulatory subunit 3 (p55, gamma) | 1.006628968 | 0.048930194 |
| SPP1 | secreted phosphoprotein 1 (osteopontin, bone sialoprotein I, early T-lymphocyte activation 1) | -1.747361776 | 0.000845446 |
| PGF | placental growth factor, vascular endothelial growth factor-related protein | -1.387482912 | 0.014012369 |
| BIRC2 | baculoviral IAP repeat-containing 2 | 1.137727113 | 0.016708782 |
| ITGB1 | integrin, beta 1 (fibronectin receptor, beta polypeptide, antigen CD29 includes MDF2, MSK12) | 0.904558067 | 0.0411008 |
| ITGAV | integrin, alpha V (vitronectin receptor, alpha polypeptide, antigen CD51) | 1.19035653 | 0.01082119 |
| LAMB1 | laminin, beta 1 | 0.992639608 | 0.031675194 |
| PAK6 | p21(CDKN1A)-activated kinase 6 | -1.282458411 | 0.010937677 |
| VCL | vinculin | 1.261421551 | 0.008152852 |
| TNR | tenascin R (restrictin, janusin) | -1.861379843 | 0.002900896 |
| CAV2 | caveolin 2 | 1.123592419 | 0.047127813 |
| **KEGG_JAK_STAT_SIGNALING_PATHWAY** |  |  |  |
| LIF | leukemia inhibitory factor (cholinergic differentiation factor) | 1.536203464 | 0.003559406 |
| CSF2 | colony stimulating factor 2 (granulocyte-macrophage) | NA | NA |
| IL6ST | interleukin 6 signal transducer (gp130, oncostatin M receptor) | 1.104173733 | 0.020177528 |
| IL20RB | interleukin 20 receptor beta | 1.034051697 | 0.023009289 |
| LIFR | leukemia inhibitory factor receptor alpha | 2.422574124 | 0.000226366 |
| OSMR | oncostatin M receptor | 2.317583381 | 0.000543184 |
| PIK3R1 | phosphoinositide-3-kinase, regulatory subunit 1 (p85 alpha) | 0.999787095 | 0.042866007 |
| PIK3R3 | phosphoinositide-3-kinase, regulatory subunit 3 (p55, gamma) | 1.006628968 | 0.048930194 |
| STAT2 | signal transducer and activator of transcription 2, 113kDa | 1.299573041 | 0.026090128 |
| SOCS3 | suppressor of cytokine signaling 3 | 1.644504309 | 0.001312385 |
| SOCS2 | suppressor of cytokine signaling 2 | 1.583441278 | 0.002460079 |
| PIM1 | pim-1 oncogene | 1.184764154 | 0.01203894 |
| **KEGG_RENAL_CELL_CARCINOMA** |  |  |  |
| VEGFB | vascular endothelial growth factor B | -1.250197889 | 0.008209675 |
| VEGFC | vascular endothelial growth factor C | -1.717939619 | 0.001511676 |
| PIK3R1 | phosphoinositide-3-kinase, regulatory subunit 1 (p85 alpha) | 0.999787095 | 0.042866007 |
| PIK3R3 | phosphoinositide-3-kinase, regulatory subunit 3 (p55, gamma) | 1.006628968 | 0.048930194 |
| PGF | placental growth factor, vascular endothelial growth factor-related protein | -1.387482912 | 0.014012369 |
| RBX1 | ring-box 1 | -0.894936836 | 0.045340549 |
| EPAS1 | endothelial PAS domain protein 1 | 2.4268684 | 8.157E-05 |
| PAK6 | p21(CDKN1A)-activated kinase 6 | -1.282458411 | 0.010937677 |
| **KEGG_MTOR_SIGNALING_PATHWAY** |  |  |  |
| VEGFB | vascular endothelial growth factor B | -1.250197889 | 0.008209675 |
| VEGFC | vascular endothelial growth factor C | -1.717939619 | 0.001511676 |
| PIK3R1 | phosphoinositide-3-kinase, regulatory subunit 1 (p85 alpha) | 0.999787095 | 0.042866007 |
| PIK3R3 | phosphoinositide-3-kinase, regulatory subunit 3 (p55, gamma) | 1.006628968 | 0.048930194 |
| PGF | placental growth factor, vascular endothelial growth factor-related protein | -1.387482912 | 0.014012369 |
| ULK2 | unc-51-like kinase 2 (C. elegans) | 1.306342148 | 0.02102742 |
| CAB39L | calcium binding protein 39-like | 1.128881852 | 0.035918877 |
| **BIOCARTA_CTCF_PATHWAY** |  |  |  |
| TGFBR2 | transforming growth factor, beta receptor II (70/80kDa) | 0.88481149 | 0.045336518 |
| PIK3R1 | phosphoinositide-3-kinase, regulatory subunit 1 (p85 alpha) | 0.999787095 | 0.042866007 |
| MDM2 | Mdm2, transformed 3T3 cell double minute 2, p53 binding protein (mouse) | 0.973982046 | 0.030622464 |
| CDKN2A | cyclin-dependent kinase inhibitor 2A (melanoma, p16, inhibits CDK4) | -1.505542503 | 0.003536159 |
| TGFBR3 | transforming growth factor, beta receptor III (betaglycan, 300kDa) | 1.27878804 | 0.02764767 |
| **KEGG_B_CELL_RECEPTOR_SIGNALING_PATHWAY** |  |  |  |
| NFKBIA | nuclear factor of kappa light polypeptide gene enhancer in B-cells inhibitor, alpha | 1.230827121 | 0.008576939 |
| PIK3R1 | phosphoinositide-3-kinase, regulatory subunit 1 (p85 alpha) | 0.999787095 | 0.042866007 |
| PIK3R3 | phosphoinositide-3-kinase, regulatory subunit 3 (p55, gamma) | 1.006628968 | 0.048930194 |
| FOS | v-fos FBJ murine osteosarcoma viral oncogene homolog | 1.532413004 | 0.002073657 |
| NFATC4 | nuclear factor of activated T-cells, cytoplasmic, calcineurin-dependent 4 | 1.112630511 | 0.046243876 |
| RASGRP3 | RAS guanyl releasing protein 3 (calcium and DAG-regulated) | -1.732132427 | 0.001917594 |
| NFKBIE | nuclear factor of kappa light polypeptide gene enhancer in B-cells inhibitor, epsilon | 1.391330396 | 0.008161975 |
| CD22 | CD22 molecule | 0.984203024 | 0.035081253 |
| **BIOCARTA_PPARA_PATHWAY** |  |  |  |
| NFKBIA | nuclear factor of kappa light polypeptide gene enhancer in B-cells inhibitor, alpha | 1.230827121 | 0.008576939 |
| PIK3R1 | phosphoinositide-3-kinase, regulatory subunit 1 (p85 alpha) | 0.999787095 | 0.042866007 |
| PRKAR2B | protein kinase, cAMP-dependent, regulatory, type II, beta | 1.763187708 | 0.003975956 |
| ME1 | malic enzyme 1, NADP(+)-dependent, cytosolic | -1.063193883 | 0.022047427 |
| APOA2 | apolipoprotein A-II | -0.965857809 | 0.040905412 |
| NRIP1 | nuclear receptor interacting protein 1 | 1.40385058 | 0.010182501 |
| NCOA1 | nuclear receptor coactivator 1 | 1.128929176 | 0.032997794 |
| **BIOCARTA_RANKL_PATHWAY** |  |  |  |
| FOS | v-fos FBJ murine osteosarcoma viral oncogene homolog | 1.532413004 | 0.002073657 |
| EIF2AK2 | eukaryotic translation initiation factor 2-alpha kinase 2 | 1.141346002 | 0.035182513 |
| FOSL1 | FOS-like antigen 1 | -1.929438791 | 0.000448667 |
| FOSL2 | FOS-like antigen 2 | -1.217798765 | 0.02443926 |
| **KEGG_PEROXISOME** |  |  |  |
| ABCD3 | ATP-binding cassette, sub-family D (ALD), member 3 | 0.974323895 | 0.038760725 |
| GSTK1 | glutathione S-transferase kappa 1 | -1.699953481 | 0.001446549 |
| PMVK | phosphomevalonate kinase | -1.35734151 | 0.007512591 |
| MLYCD | malonyl-CoA decarboxylase | 0.917886397 | 0.047465328 |
| CROT | carnitine O-octanoyltransferase | 1.076197982 | 0.026888045 |
| ECH1 | enoyl Coenzyme A hydratase 1, peroxisomal | -0.894851396 | 0.045491178 |
| PAOX | polyamine oxidase (exo-N4-amino) | -2.007771686 | 0.001979148 |
| PEX6 | peroxisomal biogenesis factor 6 | 0.886663604 | 0.047822706 |
| **KEGG_CELL_CYCLE** |  |  |  |
| MDM2 | Mdm2, transformed 3T3 cell double minute 2, p53 binding protein (mouse) | 0.973982046 | 0.030622464 |
| CDKN2A | cyclin-dependent kinase inhibitor 2A (melanoma, p16, inhibits CDK4) | -1.505542503 | 0.003536159 |
| RBX1 | ring-box 1 | -0.894936836 | 0.045340549 |
| CDC27 | cell division cycle 27 | 1.637207727 | 0.005239304 |
| GADD45B | growth arrest and DNA-damage-inducible, beta | 1.464315445 | 0.003277579 |
| CDK7 | cyclin-dependent kinase 7 (MO15 homolog, Xenopus laevis, cdk-activating kinase) | 1.272394049 | 0.016148806 |
| RBL2 | retinoblastoma-like 2 (p130) | 1.11737455 | 0.04025159 |
| YWHAE | tyrosine 3-monooxygenase/tryptophan 5-monooxygenase activation protein, epsilon polypeptide | 0.88295819 | 0.047893073 |
| ATR | ataxia telangiectasia and Rad3 related | 1.211182518 | 0.020096103 |
| TTK | TTK protein kinase | 1.009911153 | 0.039811481 |
| **KEGG_NUCLEOTIDE_EXCISION_REPAIR** |  |  |  |
| RBX1 | ring-box 1 | -0.894936836 | 0.045340549 |
| POLE4 | polymerase (DNA-directed), epsilon 4 (p12 subunit) | 1.012767041 | 0.034025561 |
| CUL4B | cullin 4B | 1.059755076 | 0.037360698 |
| CDK7 | cyclin-dependent kinase 7 (MO15 homolog, Xenopus laevis, cdk-activating kinase) | 1.272394049 | 0.016148806 |
| ERCC1 | excision repair cross-complementing rodent repair deficiency, complementation group 1 (includes overlapping antisense sequence) | -1.084409141 | 0.019310992 |
| ERCC5 | excision repair cross-complementing rodent repair deficiency, complementation group 5 (xeroderma pigmentosum, complementation group G (Cockayne syndrome)) | 1.013651122 | 0.030147534 |
| **KEGG_ECM_RECEPTOR_INTERACTION** |  |  |  |
| SPP1 | secreted phosphoprotein 1 (osteopontin, bone sialoprotein I, early T-lymphocyte activation 1) | -1.747361776 | 0.000845446 |
| ITGB1 | integrin, beta 1 (fibronectin receptor, beta polypeptide, antigen CD29 includes MDF2, MSK12) | 0.904558067 | 0.0411008 |
| ITGAV | integrin, alpha V (vitronectin receptor, alpha polypeptide, antigen CD51) | 1.19035653 | 0.01082119 |
| LAMB1 | laminin, beta 1 | 0.992639608 | 0.031675194 |
| TNR | tenascin R (restrictin, janusin) | -1.861379843 | 0.002900896 |
| DAG1 | dystroglycan 1 (dystrophin-associated glycoprotein 1) | 0.90619327 | 0.042209074 |
| SDC2 | syndecan 2 (heparan sulfate proteoglycan 1, cell surface-associated, fibroglycan) | 2.544456446 | 6.31477E-05 |
| CD47 | CD47 molecule | 1.029458737 | 0.02556912 |
| **BIOCARTA_BCELLSURVIVAL_PATHWAY** |  |  |  |
| PIK3R1 | phosphoinositide-3-kinase, regulatory subunit 1 (p85 alpha) | 0.999787095 | 0.042866007 |
| FOS | v-fos FBJ murine osteosarcoma viral oncogene homolog | 1.532413004 | 0.002073657 |
| ITGB1 | integrin, beta 1 (fibronectin receptor, beta polypeptide, antigen CD29 includes MDF2, MSK12) | 0.904558067 | 0.0411008 |
| CASP7 | caspase 7, apoptosis-related cysteine peptidase | 0.913615586 | 0.046771765 |
| **KEGG_OTHER_GLYCAN_DEGRADATION** |  |  |  |
| GLB1 | galactosidase, beta 1 | 0.924549075 | 0.038631767 |
| HEXB | hexosaminidase B (beta polypeptide) | 0.971268027 | 0.032155458 |
| MAN2B1 | mannosidase, alpha, class 2B, member 1 | 1.121448945 | 0.016019693 |
| NEU2 | sialidase 2 (cytosolic sialidase) | -1.024028524 | 0.03238475 |
| **KEGG_HYPERTROPHIC_CARDIOMYOPATHY_HCM** |  |  |  |
| ITGB1 | integrin, beta 1 (fibronectin receptor, beta polypeptide, antigen CD29 includes MDF2, MSK12) | 0.904558067 | 0.0411008 |
| ITGAV | integrin, alpha V (vitronectin receptor, alpha polypeptide, antigen CD51) | 1.19035653 | 0.01082119 |
| CACNB3 | calcium channel, voltage-dependent, beta 3 subunit | -0.948256393 | 0.039457046 |
| DAG1 | dystroglycan 1 (dystrophin-associated glycoprotein 1) | 0.90619327 | 0.042209074 |
| EMD | emerin (Emery-Dreifuss muscular dystrophy) | -1.218671263 | 0.010333385 |
| TNNC1 | troponin C type 1 (slow) | -0.992197171 | 0.048625529 |
| TNNT2 | troponin T type 2 (cardiac) | -1.437966184 | 0.003791724 |
| TPM4 | tropomyosin 4 | 0.983624364 | 0.029482806 |
| **KEGG_TYPE_II_DIABETES_MELLITUS** |  |  |  |
| PIK3R1 | phosphoinositide-3-kinase, regulatory subunit 1 (p85 alpha) | 0.999787095 | 0.042866007 |
| PIK3R3 | phosphoinositide-3-kinase, regulatory subunit 3 (p55, gamma) | 1.006628968 | 0.048930194 |
| SOCS3 | suppressor of cytokine signaling 3 | 1.644504309 | 0.001312385 |
| SOCS2 | suppressor of cytokine signaling 2 | 1.583441278 | 0.002460079 |
| IRS1 | insulin receptor substrate 1 | -1.328420495 | 0.006201731 |
| PRKCE | protein kinase C, epsilon | 1.146490499 | 0.045578986 |
| **BIOCARTA_ARF_PATHWAY** |  |  |  |
| PIK3R1 | phosphoinositide-3-kinase, regulatory subunit 1 (p85 alpha) | 0.999787095 | 0.042866007 |
| MDM2 | Mdm2, transformed 3T3 cell double minute 2, p53 binding protein (mouse) | 0.973982046 | 0.030622464 |
| CDKN2A | cyclin-dependent kinase inhibitor 2A (melanoma, p16, inhibits CDK4) | -1.505542503 | 0.003536159 |
| POLR1B | polymerase (RNA) I polypeptide B, 128kDa | -1.135878759 | 0.016378481 |
| **KEGG_EPITHELIAL_CELL_SIGNALING_IN_HELICOBACTER_PYLORI_INFECTION** |  |  |  |
| CCL5 | chemokine (C-C motif) ligand 5 | NA | NA |
| CXCL1 | chemokine (C-X-C motif) ligand 1 (melanoma growth stimulating activity, alpha) | 3.515131665 | 7.46425E-06 |
| NFKBIA | nuclear factor of kappa light polypeptide gene enhancer in B-cells inhibitor, alpha | 1.230827121 | 0.008576939 |
| MAP2K4 | mitogen-activated protein kinase kinase 4 | 1.011406469 | 0.031394709 |
| HBEGF | heparin-binding EGF-like growth factor | 1.007160902 | 0.02596326 |
| ADAM17 | ADAM metallopeptidase domain 17 (tumor necrosis factor, alpha, converting enzyme) | 1.825737043 | 0.00173848 |
| ADAM10 | ADAM metallopeptidase domain 10 | 1.177236402 | 0.014761754 |
| **BIOCARTA_ETS_PATHWAY** |  |  |  |
| CSF1 | colony stimulating factor 1 (macrophage) | 2.008134032 | 0.000324265 |
| FOS | v-fos FBJ murine osteosarcoma viral oncogene homolog | 1.532413004 | 0.002073657 |
| RBL2 | retinoblastoma-like 2 (p130) | 1.11737455 | 0.04025159 |
| ETV3 | ets variant gene 3 | 1.02648019 | 0.037660126 |
| **KEGG_METABOLISM_OF_XENOBIOTICS_BY_CYTOCHROME_P450** |  |  |  |
| GSTK1 | glutathione S-transferase kappa 1 | -1.699953481 | 0.001446549 |
| MGST1 | microsomal glutathione S-transferase 1 | 1.403642478 | 0.00463756 |
| MGST2 | microsomal glutathione S-transferase 2 | -1.939343266 | 0.000474453 |
| MGST3 | microsomal glutathione S-transferase 3 | -1.711648887 | 0.001074239 |
| ALDH3A1 | aldehyde dehydrogenase 3 family, memberA1 | -1.770886631 | 0.003651685 |
| CYP1A1 | cytochrome P450, family 1, subfamily A, polypeptide 1 | -1.067709822 | 0.024438746 |
| CYP2S1 | cytochrome P450, family 2, subfamily S, polypeptide 1 | -0.93066616 | 0.038157664 |
| **KEGG_PANCREATIC_CANCER** |  |  |  |
| VEGFB | vascular endothelial growth factor B | -1.250197889 | 0.008209675 |
| VEGFC | vascular endothelial growth factor C | -1.717939619 | 0.001511676 |
| TGFBR2 | transforming growth factor, beta receptor II (70/80kDa) | 0.88481149 | 0.045336518 |
| PIK3R1 | phosphoinositide-3-kinase, regulatory subunit 1 (p85 alpha) | 0.999787095 | 0.042866007 |
| PIK3R3 | phosphoinositide-3-kinase, regulatory subunit 3 (p55, gamma) | 1.006628968 | 0.048930194 |
| PGF | placental growth factor, vascular endothelial growth factor-related protein | -1.387482912 | 0.014012369 |
| CDKN2A | cyclin-dependent kinase inhibitor 2A (melanoma, p16, inhibits CDK4) | -1.505542503 | 0.003536159 |
| **KEGG_DILATED_CARDIOMYOPATHY** |  |  |  |
| ITGB1 | integrin, beta 1 (fibronectin receptor, beta polypeptide, antigen CD29 includes MDF2, MSK12) | 0.904558067 | 0.0411008 |
| ITGAV | integrin, alpha V (vitronectin receptor, alpha polypeptide, antigen CD51) | 1.19035653 | 0.01082119 |
| CACNB3 | calcium channel, voltage-dependent, beta 3 subunit | -0.948256393 | 0.039457046 |
| DAG1 | dystroglycan 1 (dystrophin-associated glycoprotein 1) | 0.90619327 | 0.042209074 |
| EMD | emerin (Emery-Dreifuss muscular dystrophy) | -1.218671263 | 0.010333385 |
| TNNC1 | troponin C type 1 (slow) | -0.992197171 | 0.048625529 |
| TNNT2 | troponin T type 2 (cardiac) | -1.437966184 | 0.003791724 |
| TPM4 | tropomyosin 4 | 0.983624364 | 0.029482806 |
| **KEGG_FRUCTOSE_AND_MANNOSE_METABOLISM** |  |  |  |
| MTMR1 | myotubularin related protein 1 | 1.226309698 | 0.024707945 |
| MTMR6 | myotubularin related protein 6 | 1.11675421 | 0.023773542 |
| KHK | ketohexokinase (fructokinase) | -1.115904879 | 0.028925633 |
| PFKFB3 | 6-phosphofructo-2-kinase/fructose-2,6-biphosphatase 3 | 1.460986036 | 0.004402713 |
| SORD | sorbitol dehydrogenase | -0.979412853 | 0.032591064 |
| **KEGG_PYRIMIDINE_METABOLISM** |  |  |  |
| POLE4 | polymerase (DNA-directed), epsilon 4 (p12 subunit) | 1.012767041 | 0.034025561 |
| POLR1B | polymerase (RNA) I polypeptide B, 128kDa | -1.135878759 | 0.016378481 |
| POLR2H | polymerase (RNA) II (DNA directed) polypeptide H | -0.944306445 | 0.034725364 |
| PRIM1 | primase, polypeptide 1, 49kDa | 1.03032361 | 0.026531892 |
| NME4 | non-metastatic cells 4, protein expressed in | -1.298103367 | 0.00691251 |
| NME6 | non-metastatic cells 6, protein expressed in (nucleoside-diphosphate kinase) | -1.518539181 | 0.002669182 |
| CDA | cytidine deaminase | -0.987491942 | 0.03289615 |
| TK1 | thymidine kinase 1, soluble | -1.123814676 | 0.036796819 |
| **BIOCARTA_CALCINEURIN_PATHWAY** |  |  |  |
| NFATC4 | nuclear factor of activated T-cells, cytoplasmic, calcineurin-dependent 4 | 1.112630511 | 0.046243876 |
| MARCKS | myristoylated alanine-rich protein kinase C substrate | 1.012663287 | 0.02479483 |
| GNAQ | guanine nucleotide binding protein (G protein), q polypeptide | 1.320530401 | 0.011912157 |
| SP3 | Sp3 transcription factor | 1.188013471 | 0.022063175 |
| **BIOCARTA_NFAT_PATHWAY** |  |  |  |
| LIF | leukemia inhibitory factor (cholinergic differentiation factor) | 1.536203464 | 0.003559406 |
| PIK3R1 | phosphoinositide-3-kinase, regulatory subunit 1 (p85 alpha) | 0.999787095 | 0.042866007 |
| NFATC4 | nuclear factor of activated T-cells, cytoplasmic, calcineurin-dependent 4 | 1.112630511 | 0.046243876 |
| PRKAR2B | protein kinase, cAMP-dependent, regulatory, type II, beta | 1.763187708 | 0.003975956 |
| HBEGF | heparin-binding EGF-like growth factor | 1.007160902 | 0.02596326 |
| EDN1 | endothelin 1 | 1.383568505 | 0.01246535 |
| **KEGG_NEUROTROPHIN_SIGNALING_PATHWAY** |  |  |  |
| SORT1 | sortilin 1 | 1.380701136 | 0.004838616 |
| NFKBIA | nuclear factor of kappa light polypeptide gene enhancer in B-cells inhibitor, alpha | 1.230827121 | 0.008576939 |
| PIK3R1 | phosphoinositide-3-kinase, regulatory subunit 1 (p85 alpha) | 0.999787095 | 0.042866007 |
| PIK3R3 | phosphoinositide-3-kinase, regulatory subunit 3 (p55, gamma) | 1.006628968 | 0.048930194 |
| RPS6KA5 | ribosomal protein S6 kinase, 90kDa, polypeptide 5 | 0.93686799 | 0.041860294 |
| NFKBIE | nuclear factor of kappa light polypeptide gene enhancer in B-cells inhibitor, epsilon | 1.391330396 | 0.008161975 |
| YWHAE | tyrosine 3-monooxygenase/tryptophan 5-monooxygenase activation protein, epsilon polypeptide | 0.88295819 | 0.047893073 |
| IRS1 | insulin receptor substrate 1 | -1.328420495 | 0.006201731 |
| FRS2 | fibroblast growth factor receptor substrate 2 | 0.989669967 | 0.049209494 |
| **KEGG_AXON_GUIDANCE** |  |  |  |
| ITGB1 | integrin, beta 1 (fibronectin receptor, beta polypeptide, antigen CD29 includes MDF2, MSK12) | 0.904558067 | 0.0411008 |
| NFATC4 | nuclear factor of activated T-cells, cytoplasmic, calcineurin-dependent 4 | 1.112630511 | 0.046243876 |
| PAK6 | p21(CDKN1A)-activated kinase 6 | -1.282458411 | 0.010937677 |
| ARHGEF12 | Rho guanine nucleotide exchange factor (GEF) 12 | 1.045940749 | 0.024952229 |
| EFNA1 | ephrin-A1 | -1.377495279 | 0.005086288 |
| EFNA4 | ephrin-A4 | -1.061506506 | 0.030669969 |
| NGEF | neuronal guanine nucleotide exchange factor | -1.575991738 | 0.003098281 |
| NRP1 | neuropilin 1 | 2.014254283 | 0.00162375 |
| RHOD | ras homolog gene family, member D | -0.877300866 | 0.048496645 |
| **KEGG_SPHINGOLIPID_METABOLISM** |  |  |  |
| GLB1 | galactosidase, beta 1 | 0.924549075 | 0.038631767 |
| NEU2 | sialidase 2 (cytosolic sialidase) | -1.024028524 | 0.03238475 |
| PPAP2B | phosphatidic acid phosphatase type 2B | -1.57029214 | 0.004586711 |
| ASAH2 | N-acylsphingosine amidohydrolase (non-lysosomal ceramidase) 2 | 1.269676765 | 0.017235974 |
| SGPP1 | sphingosine-1-phosphate phosphatase 1 | 1.703490002 | 0.001743798 |
| **BIOCARTA_NTHI_PATHWAY** |  |  |  |
| TGFBR2 | transforming growth factor, beta receptor II (70/80kDa) | 0.88481149 | 0.045336518 |
| NFKBIA | nuclear factor of kappa light polypeptide gene enhancer in B-cells inhibitor, alpha | 1.230827121 | 0.008576939 |
| MAP2K6 | mitogen-activated protein kinase kinase 6 | -0.976346687 | 0.046764851 |
| TLR2 | toll-like receptor 2 | 1.154309315 | 0.043522142 |
| **BIOCARTA_EIF2_PATHWAY** |  |  |  |
| EIF2AK2 | eukaryotic translation initiation factor 2-alpha kinase 2 | 1.141346002 | 0.035182513 |
| EIF2S2 | eukaryotic translation initiation factor 2, subunit 2 beta, 38kDa | -1.202444475 | 0.021486018 |
| EIF2AK4 | eukaryotic translation initiation factor 2 alpha kinase 4 | 1.078834185 | 0.029955583 |
| **KEGG_T_CELL_RECEPTOR_SIGNALING_PATHWAY** |  |  |  |
| CSF2 | colony stimulating factor 2 (granulocyte-macrophage) | NA | NA |
| NFKBIA | nuclear factor of kappa light polypeptide gene enhancer in B-cells inhibitor, alpha | 1.230827121 | 0.008576939 |
| PIK3R1 | phosphoinositide-3-kinase, regulatory subunit 1 (p85 alpha) | 0.999787095 | 0.042866007 |
| PIK3R3 | phosphoinositide-3-kinase, regulatory subunit 3 (p55, gamma) | 1.006628968 | 0.048930194 |
| FOS | v-fos FBJ murine osteosarcoma viral oncogene homolog | 1.532413004 | 0.002073657 |
| NFATC4 | nuclear factor of activated T-cells, cytoplasmic, calcineurin-dependent 4 | 1.112630511 | 0.046243876 |
| PAK6 | p21(CDKN1A)-activated kinase 6 | -1.282458411 | 0.010937677 |
| NFKBIE | nuclear factor of kappa light polypeptide gene enhancer in B-cells inhibitor, epsilon | 1.391330396 | 0.008161975 |
| **KEGG_SMALL_CELL_LUNG_CANCER** |  |  |  |
| NFKBIA | nuclear factor of kappa light polypeptide gene enhancer in B-cells inhibitor, alpha | 1.230827121 | 0.008576939 |
| PIK3R1 | phosphoinositide-3-kinase, regulatory subunit 1 (p85 alpha) | 0.999787095 | 0.042866007 |
| PIK3R3 | phosphoinositide-3-kinase, regulatory subunit 3 (p55, gamma) | 1.006628968 | 0.048930194 |
| BIRC2 | baculoviral IAP repeat-containing 2 | 1.137727113 | 0.016708782 |
| ITGB1 | integrin, beta 1 (fibronectin receptor, beta polypeptide, antigen CD29 includes MDF2, MSK12) | 0.904558067 | 0.0411008 |
| ITGAV | integrin, alpha V (vitronectin receptor, alpha polypeptide, antigen CD51) | 1.19035653 | 0.01082119 |
| LAMB1 | laminin, beta 1 | 0.992639608 | 0.031675194 |
